# Reversible and Massive Structural Transformation in Meltable Cyanido‐bridged Coordination Polymer Crystals

**DOI:** 10.1002/chem.202502640

**Published:** 2025-10-25

**Authors:** Yuudai Iwai, Saaya Kimura, Manabu Nakaya, Takanori Nakane, Akihiro Kawamoto, Genji Kurisu, Yuta Tsuji, Kenji Hirai, Koji Kimoto, Ovidiu Cretu, Fumitaka Takeiri, Kunihisa Sugimoto, Benjamin Le Ouay, Masaaki Ohba, Ryo Ohtani

**Affiliations:** ^1^ Department of Chemistry, Faculty of Science Kyushu University 744 Motooka Nishi‐ku Fukuoka 819‐0395 Japan; ^2^ Department of Chemistry and Biological Science, Faculty of Science Josai University 1‐1 Keyakidai Sakado Saitama 350‐0295 Japan; ^3^ Institute for Protein Research The University of Osaka 3‐2 Yamadaoka Suita Osaka 565–0871 Japan; ^4^ JEOL YOKOGUSHI Research Alliance Laboratories Graduate School of Frontier Biosciences The University of Osaka 1–3 Yamadaoka Suita Osaka 565–0871 Japan; ^5^ Faculty of Engineering Sciences Kyushu University Kasuga Fukuoka 816–8580 Japan; ^6^ Division of Photonics and Optical Science Research Institute for Electronic Science (RIES) Hokkaido University North 20 West 10, Kita ward Sapporo Hokkaido 001–0020 Japan; ^7^ Center for Basic Research on Materials 1‐1 Namiki Tsukuba Ibaraki 305‐0044 Japan; ^8^ Department of Chemistry Kindai University 3‐4‐1 Kowakae Higashi‐osaka Osaka 577–8502 Japan

**Keywords:** coordination polymer, melting, metal cyanido

## Abstract

Cyanido (CN^−^)‐bridged coordination polymers (CP) have been extensively studied as molecular‐based functional materials. However, synthesizing 3D compounds composed only of metal ions and CN^−^—without bulky organic groups—and that melt before decomposing remains a considerable challenge. This difficulty arises because CN^−^ strongly interconnect metal ions, forming rigid, dense frameworks with high melting points. In this study, we successfully synthesized a melting composite consisting of 3D KCd[Cu(CN)_2_]_3_ and 2D K_2_Cu_3_(CN)_5_ by dehydrating K_2_Cd(H_2_O)Cu_4_(CN)_8_·1.5H_2_O. Remarkably, nanodomains of these two compounds coexisted within single particles, allowing their crystal structures to be independently determined by 3D electron diffraction (MicroED) of the resulting powders. Each compound melted at its respective melting point, around 559 K. Notably, the melting point of KCd[Cu(CN)_2_]_3_ is unusually low for a 3D dense coordination framework. This atypically low melting point results from a combination of crystalline surface effects, and the entropy contribution of the dynamic, labile two‐coordinate Cu centers in the framework. Additionally, we demonstrated a reversible transformation between the dehydrated mixture and the hydrated parent compound through exposure to water vapor, highlighting the dynamic and responsive nature of these CN^−^‐based solid‐state materials.

## Graphical abstract

Melting crystals containing separated domains of KCd[Cu(CN)_2_]_3_ and K_2_Cu_3_(CN)_5_ were obtained via simple dehydration of K_2_Cd(H_2_O)Cu_4_(CN)_8_·1.5H_2_O. Their low melting point of 559 K resulted from the combined effects of strong crystal‐interface interactions and low‐coordinate Cu nodes.

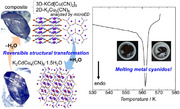

## 1 Introduction

Phase transitions in inorganic solid‐state materials present new opportunities for their development and applications. Over the past decade, the melting behavior of coordination frameworks—such as coordination polymers (CPs) and metal–organic frameworks (MOFs)—has been extensively studied, focusing not only on fundamental insights but also on practical material applications.^[^ ^1^, ^2^, ^3^ ^]^ Among these, the most thoroughly investigated melting MOFs are the zeolitic imidazolate framework series,^[^ ^4^, ^5^, ^6^, ^7^ ^]^ followed by phosphoric acid‐based CPs^[^ ^8^, ^9^, ^10^, ^11^ ^]^ and carboxylic acid‐bridged MOFs.^[^ ^12^ ^]^ These studies have indicated that using bulky ligands with delocalized charges and/or long aliphatic chains was effective for producing melting MOFs because this promotes weak coordination bonds between metal ions and ligands and/or increases entropy through kinetic, conformational, and configurational effects. In this context, small ligands such as cyanido (CN^−^), which possess a strong dipole, completely defy these design principles. Consequently, melting 3D CN^−^‐bridged CPs have not been observed, and their synthesis remains a considerable challenge because CN^−^ tend to decompose at relatively low temperatures—that is, their decomposition temperature is generally lower than their melting point.

CN^−^‐bridged CPs exhibit a wide range of functionalities, including magnetic,^[^ ^13^, ^14^, ^15^, ^16^ ^]^ electronic,^[^ ^17^, ^18^, ^19^, ^20^ ^]^ and adsorption properties.^[^ ^21^, ^22^, ^23^ ^]^ Prussian blue, first synthesized in 1704 and historically used as a blue pigment, is often considered the prototype of CPs and MOFs. The relationship between structure and properties—encompassing metal species as well as the dimensionality and topology of frameworks—has long been a key focus for advancing CN^−^‐based solid‐state materials. More recently, the structural complexity of seemingly simple CN^−^‐bridged frameworks has been explored^[^ ^24^, ^25^ ^]^; correlated disorder in the Prussian blue analog (PBA) lattice and the glassy phase of PBA were identified, further improving our understanding of defects for material applications. Since the 20th century, researchers have synthesized numerous related compounds by combining metal ions with cyanometallate units such as [M(CN)_6_]*^n^* ^−^ (*n* = 3, 4), [M(CN)_8_]*^n^* ^−^ (*n* = 3, 4), [M(CN)_4_]^2−^, and [M(CN)_2_]^−^ (Figure S1).^[^ ^26^, ^27^, ^28^, ^29^ ^]^ These efforts have revealed intriguing properties beyond melting, including negative thermal expansion^[^ ^30^, ^31^, ^32^, ^33^ ^]^ and negative linear compression.^[^ ^32^, ^34^ ^]^ This highlights the need for new synthetic strategies to explore yet‐undiscovered 3D‐melting metal–CN^−^ frameworks.

In this study, we demonstrated the synthesis of the 3D compound KCd[Cu(CN)_2_]_3_ and its melting behavior, attributed to distinctive crystal‐interface and structural effects. KCd[Cu(CN)_2_]_3_ is an analog of the well‐known wine‐rack‐type KCd[M(CN)_2_]_3_ (M = Au and Ag)^[^ ^31^, ^32^ ^]^ and represents the first melting 3D metal–CN^−^ framework reported. This compound was synthesized through a substantial structural transformation and phase separation of K_2_Cd(H_2_O)Cu_4_(CN)_8_·1.5H_2_O via dehydration. Uniquely, a single dehydrated particle contained not only KCd[Cu(CN)_2_]_3_ but also minor amounts of a melting 2D K_2_Cu_3_(CN)_5_ phase. Detailed investigation of the phase transition showed that each compound in the composite melted at its own melting point, approximately 559 K. The relatively low melting points, compared to other 3D‐melting MOFs, resulted from surface interactions between the crystalline nanodomains of KCd[Cu(CN)_2_]_3_ and K_2_Cu_3_(CN)_5._ Additionally, the mixture could be reversibly converted back to the parent K_2_Cd(H_2_O)Cu_4_(CN)_8_·1.5H_2_O through water adsorption. These unusual dynamic behaviors were further attributed to the flexibility of the low‐coordinate copper nodes, as supported by molecular dynamics simulations.

## 2 Results and Discussion

**Figure 1:**
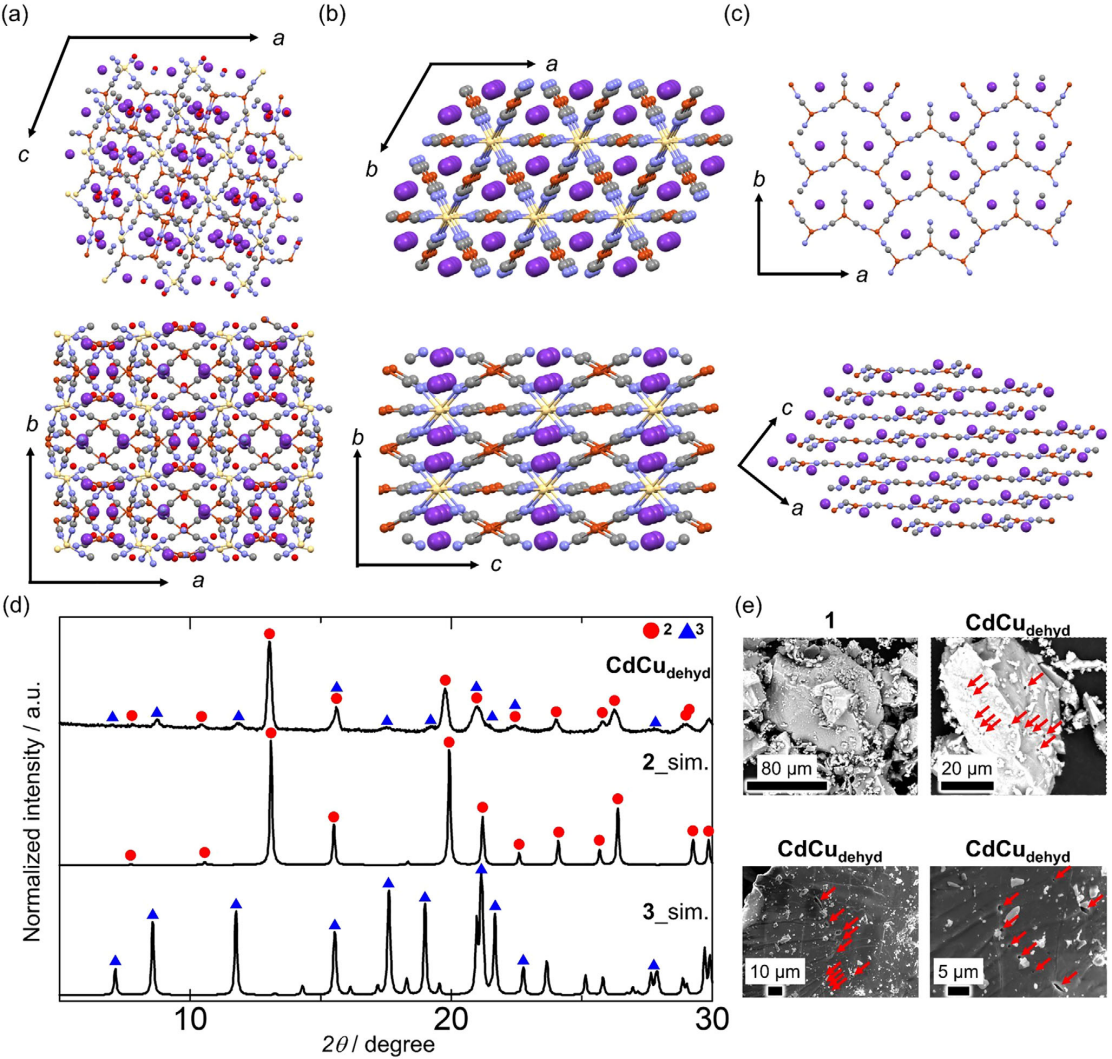
Crystal structures of a) **1**, b) **2,** and c) **3**. Color code: light yellow; Cd, orange; Cu, light blue; N, gray; C, red; O, purple; K. d) powder X‐ray diffraction (PXRD) pattern of **CdCu_dehyd_** and simulation patterns of **2** and **3**. λ = 1.08 Å. e) Scanning electron microscopy images of **1** and **CdCu_dehyd_**. Red allows indicate small holes on the surface.

Colorless block single crystals of K_2_Cd(H_2_O)Cu_4_(CN)_8_·1.5H_2_O^33^ (**1**) were obtained using aqueous solutions of K_2_Cd(CN)_4_, K[Ntf_2_] (Ntf_2_ = bis(trifluoromethanesulfonyl)imide), and CuCN (Figure 1a, 1e and Table S1). This compound has a 3D CN^−^‐bridged anionic framework. The Cu ions are planar and three‐coordinated, with a bond distance to C/N in the first coordination sphere of approximately 1.9 Å. The Cd ions form three‐way bipyramidal nodes coordinated by four CN^−^ groups and one water molecule. The bond lengths were measured as 2.175 Å for Cd─N and 2.559 Å for Cd─O(H_2_O). Infrared (IR) spectroscopy revealed a stretching vibration peak of the CN group at 2112 cm^−1^ and characteristic peaks from water molecules around 3600 cm^−1^ (Figure S1). Thermogravimetric analysis (TGA) of **1** indicated the loss of both crystalline and coordinated water molecules up to 450 K (Figure S2). The dehydrated sample (**CdCu_dehyd_**) remained stable up to approximately 600 K. The powder X‐ray diffraction (PXRD) pattern of **CdCu_dehyd_**—obtained by heating **1** at 450 K—differed from that of the original compound (Figure S3).

We found that **CdCu_dehyd_** was a mixture of two crystalline CN^−^‐bridged CPs—KCd[Cu(CN)_2_]_3_ (**2**) and K_2_Cu_3_(CN)_5_ (**3**)^[^ ^34^ ^]^—based on 3D electron diffraction (MicroED) measurements of the dehydrated powder at 79 K (Figure 1b, 1c and S4, S5 and Tables S2–S4). Compound **2** crystallizes in the *P*‐31*m* space group. The Cd ions adopt an octahedral geometry by bridging with slightly bent Cu(CN)_2_ units, forming a 3D wine‐rack‐type framework with K^+^ ions located in the framework's interstitial spaces. Notably, **2** is isostructural with its well‐known analogs KCd[M(CN)_2_]_3_ (M = Ag, Au). ^[^ ^31^, ^32^ ^]^ The Cd─N bond length of 2.34 Å is comparable to those of the Ag (2.332 Å) and Au (2.331 Å) analogs. The distance between the closest Cu atoms along the *a*‐axis is 3.39 Å, slightly longer than 3.376 Å in KCd[Ag(CN)_2_]_3_ and 3.318 Å in KCd[Au(CN)_2_]_3_ (Figure S6). In contrast, compound **3** crystallizes in the *C*2/*c* space group, forming an anisotropic layered structure of [Cu_3_(CN)_5_]^2−^ with three‐coordinate Cu nodes, while K^+^ ions occupy the interlayer spaces. Compound **3** has an incomplete pentagonal network owing to a single nonbridging CN^−^ ligand. The PXRD pattern of **CdCu_dehyd_** can be interpreted as a combination of the simulated PXRD patterns of **2** and **3** (Figure 1d). Based on the peak intensity ratio, **2** is the predominant phase in **CdCu_dehyd_**. The IR spectra of **CdCu_dehyd_** exhibited CN stretching mode peaks at 2145 and 2103 cm^−1^, along with two shoulders at 2087 and 2122 cm^−1^ (Figure S1). Comparing these to the IR spectra of KCd[Ag(CN)_2_]_3_ and KCd[Au(CN)_2_]_3_, which show a CN stretching mode at 2156 cm^−1^, the peak at 2145 cm^−1^ can be attributed to **2**. Therefore, the remaining three peaks correspond to **3**, consistent with the presence of three crystallographically independent CN groups.

Interestingly, the formation of **2** and **3** through the dehydration of **1** involved extensive structural changes while preserving the overall crystal morphology, despite the appearance of small holes on the crystal surface (Figures 1d, 1e, and S7). This finding confirms the coexistence of the two crystalline domains, **2** and **3**, in a single composite particle. However, no combination of stoichiometric ratios of **2** and **3** can fully represent the formula of **1**, indicating that **CdCu_dehyd_** also contains an amorphous component. This conclusion is supported by transmission electron microscopy–energy‐dispersive X‐ray spectroscopy (TEM–EDX), which detected amorphous CuCN alongside the crystalline nanodomains of **2** and **3** (Figure S8 and Table S5). However, the IR spectra showed no peaks corresponding to CuCN (2162 cm^−1^) (Figure S1), confirming that the amount of CuCN residue in **CdCu_dehyd_** was negligible. The intracrystalline phase separation into **2** and **3** from **1** indicates that a dehydrated **1** is unstable, likely due to the pentacoordinate Cd centers within the network, and it subsequently transforms into the thermodynamically more stable hexacoordinate Cd‐based compound of **2**. Subsequently, **3** and an amorphous phase derived from the remaining components are formed sequentially.

**Figure 2:**
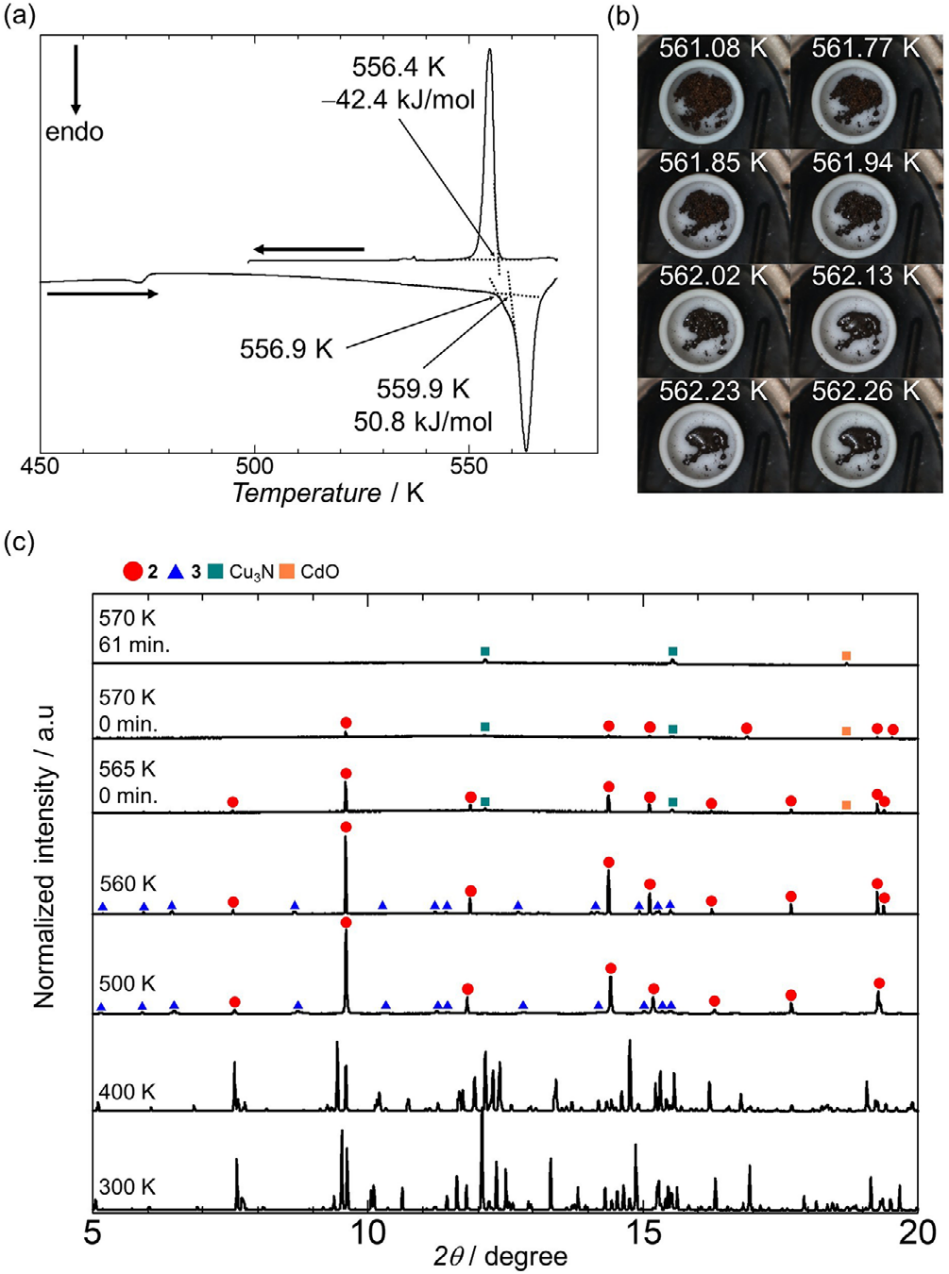
a) DSC curves of **1**, with enthalpy values calculated based on its molecular weight. b) Photographs of **CdCu_dehyd_** at 561.08–562.26 K during DSC measurements. c) VT–PXRD results of **1** during heating at 10 K/minute (λ = 0.799 585 Å). Red circles, blue triangles, green squares, and orange squares mark the diffraction peaks of **2**, **3**, Cu_3_N, and CdO, respectively.

Differential scanning calorimetry (DSC) results revealed that **CdCu_dehyd_** melted (Figure 2a, 2b). The DSC curves of **1** exhibited a small peak at 470.5 K followed by a large endothermic peak with a minor shoulder near 559 K during heating at 5 K/minute. In situ imaging showed that the first peak corresponds to dehydration forming **CdCu_dehyd_**, while the second peak indicates melting of the composite. During the subsequent cooling, an exothermic solidification peak was observed at 556.4 K.

To elucidate the melting behavior of **CdCu_dehyd_**, which contains **2** and **3**, variable‐temperature powder X‐ray diffraction (VT–PXRD) measurements were performed under vacuum (Figures 2c, S9–S18). During heating, the diffraction pattern of **3** disappeared at 565 K, consistent with its melting at this temperature, while the pattern of **2** remained but with reduced intensity (Figures 2c, S9–S11, and S18a). At 570 K, the diffraction pattern of **2** also disappeared (Figures 2c, S12, and S13), indicating that the melting temperature of **2** was higher than that of **3** in **CdCu_dehyd_**. Notably, small new peaks emerged at 12.1° and 15.5° following the melting of **3** at 565 K (marked by green squares in Figure 2c). After **2** melted at 570 K, an additional diffraction peak appeared at 18.7° (orange squares in Figures 2c and S13). The peaks at 12.1° and 15.5° and the peak at 18.7° are consistent with the formation of Cu_3_N (peaks at 12.1° and 15.5°)^[^ ^35^ ^]^ and CdO (peak at 18.7°)^[^ ^36^ ^]^ from reactions of the molten **2** and **3** with trace O_2_ and N_2_. The VT–PXRD cooling process confirmed the reversible phase transition of **2** and **3**, despite changes in peak intensity ratios owing to recrystallization and minor sample loss from side reactions. VT–IR measurements showed that the liquid phase exhibited a single CN stretching mode at 2104 cm^−1^ (Figure S19), indicating the formation of a homogeneous ionic liquid state through the breaking of CN^−^ bridges during melting. VT–PXRD measurements from 100 to 450 K additionally provided characteristic anisotropic thermal expansion behavior for **2**, yielding linear coefficients of α*_a_*_,_ *_b_* = 41.8 (16) MK^−1^ (M = 10^6^), α*_c_* = −47.1 (6) MK^−1^, and α*_V_* = 35.2 MK^−1^. A comprehensive discussion of this behavior is depicted in Figures S20, S21 and Tables S6, S7. Finally, TEM–EDX analysis also identified the presence of CdO in the sample after melting (Figure S22 and Table S8); it is noteworthy that Cu_3_N proved challenging to detect owing to interference from the instrument's copper components.

It is important to note that **2** cannot be obtained using [Cu(CN)_2_]^−^ in the synthetic protocol for KCd[M(CN)_2_]_3_ (M = Ag and Au). In contrast, although **3** was a known entity, its thermodynamic behavior had not been elucidated. Therefore, we investigated the phase transition behavior of **3** by synthesizing its powders via a reported method^[^ ^34^ ^]^ (Figure S23) and performing TG‐DTA measurements (Figure S24a). Our findings indicate that a single phase of **3** melts at 600 K, a temperature exceeding its melting point when incorporated in **CdCu_dehyd_**. The decrease in the melting points of **2** and **3** in **CdCu_dehyd_** would be partly attributed to lattice defects, tiny crystal domains, and amorphous residues which were generated by the large structural transformation via dehydration. On the other hand, this observation also implies that the distinctive low melting point of **CdCu_dehyd_** arises from a nanoscale crystal‐interface interaction between **2** and **3**. Further evidence for this melting point depression was provided by solid‐state mixing experiments involving **CdCu_dehyd_** and **3** powders. The melting temperatures of the resulting mixtures changed as the mixing ratio of **3** increased (Figure S24b and Table S9). This observation suggests that despite **2** and **3** possessing completely distinct crystal phases, the CN^−^ groups on their respective crystal surfaces interact with the metal ions of neighboring crystals during solid mixing, thereby affecting the thermodynamic properties of the composite materials.

Conventional strategies for synthesizing meltable MOFs and CPs have typically used bulky and electrodelocalized ligands, including imidazolate cations,^[^ ^4^, ^5^, ^6^, ^7^, ^37^, ^38^, ^39^ ^]^ [Ntf_2_] anions,^[^ ^40^, ^41^, ^42^ ^]^ phosphoric acid,^[^ ^8^, ^9^, ^10^, ^11^ ^]^ and methanetricarbonitrile.^[^ ^39^, ^43^, ^44^ ^]^ These larger molecular components reduce the number of coordination bonds, thereby contributing enthalpically to lower melting temperatures, and increase conformational entropy, contributing entropically. In this context, it is notable that **2**, a 3D CN^−^‐bridged framework composed only of small, anisotropic CN^−^ linkers, melts at approximately 559 K (Figures S25, S26, and Table S10). Notably, the analogous compounds, KCd[M(CN)_2_]_3_ (M = Ag and Au), do not exhibit melting behavior (Figure S27). This observation implied the role of Cu nodes in the melting of **CdCu_dehyd_**, in addition to the previously discussed crystal‐interface effects. Therefore, the unique low‐temperature melting of **2** was theoretically explored via molecular dynamics simulations to elucidate the impact of metal species at the two‐coordinate site on the dynamic behavior of the overall KCd[M(CN)_2_]_3_ network (M = Cu, Ag, and Au). Notably, the simulation results for **2** revealed a substantial fluctuation of Cu nodes, coupled with changes in their coordination number, at high temperatures in 1500 fs (Figure S28 and Supplementary movie 1). Additionally, the radial distribution function (RDF) of **2** was examined across time frames such as 0–500 fs and 500–1000 fs, and 1000–1500‐fs segments, revealing a broadening of the peak for the first coordination sphere of the Cu center with increasing time (Figure S29). These drastic changes in the coordination sphere and RDF peaks were absent in KCd[Ag(CN)_2_]_3_ and KCd[Au(CN)_2_]_3_ when subjected to the same conditions. Furthermore, the enthalpies of formation for these three compounds were confirmed to exhibit only minor differences (Table S11). Therefore, we conclude that the flexible and labile nature of the two‐coordinate Cu nodes increases the entropy change upon melting, leading to a reduced melting point (Δ*H*/Δ*S*).

**Figure 3:**
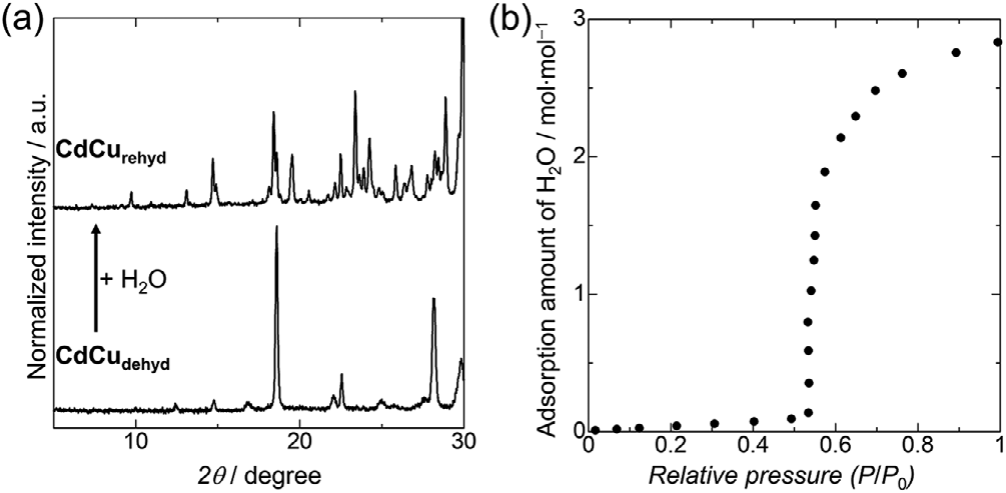
a) PXRD results showing the reversible structural transformation of **CdCu_dehyd_** to **CdCu_rehyd_** upon exposure to water vapor (λ = 1.54 Å). The powder pattern of **CdCu_rehyd_** matches that of **1**. b) Water adsorption isotherm of **CdCu_dehyd_** at 298 K.

**Scheme 1:**
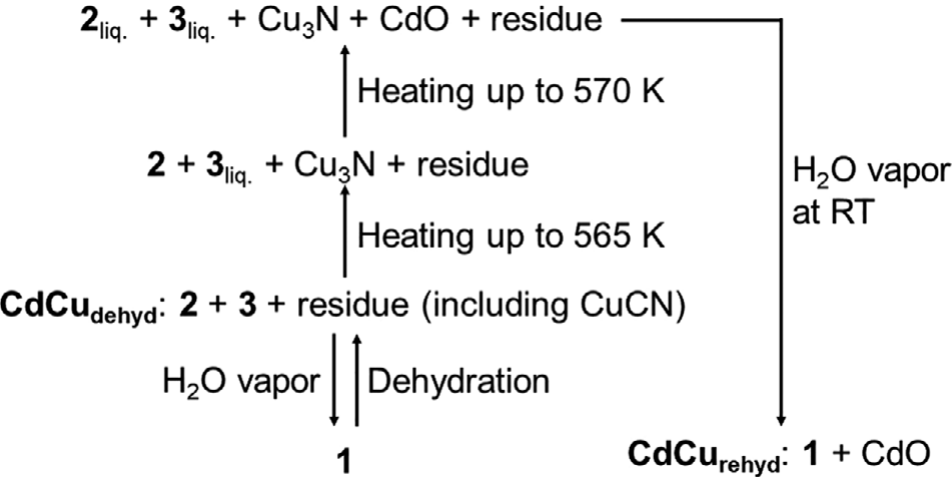
Overview of the structural conversion and phase transition of the system.

Remarkably, **CdCu_dehyd_**, comprising **2** and **3**, transformed back into **1** (**CdCu_rehyd_**) upon exposure to water vapor. Analytical data from PXRD, IR, TGA, and elemental analysis of **CdCu_rehyd_** were found to be consistent with those of precursor **1** (Figures 3a, S30, and Scheme 1), despite the minor presence of CdO detected via TEM–EDX (Figure S31 and Table S12). This distinctive rehydration behavior further substantiates that the separated nanodomains of **2** and **3** possess robust interfacial connections in each particle, allowing them to collectively react to water vapor and regenerate the parent **1** as if they constitute a single material. Importantly, the crystal morphology remained intact after rehydration (Figures S7 and S32), indicating a solid–solid transformation despite substantial structural changes. To further elucidate this reversible interconversion between **CdCu_dehyd_** and **1**, the water adsorption isotherm of **CdCu_dehyd_** was measured at room temperature. Observations indicated gate‐opening type adsorption behavior starting at approximately 0.5 relative pressure, with a total uptake of approximately 2.8 water molecules (Figure 3b). The manifestation of this gate‐opening behavior strongly supports the occurrence of a substantial structural transformation during the interconversion of **CdCu_dehyd_** and **1**.

## 3 Conclusion

Simple dehydration of K_2_Cd(H_2_O)Cu_4_(CN)_8_∙1.5H_2_O resulted in unique melting crystals containing discrete domains of KCd[Cu(CN)_2_]_3_ and K_2_Cu_3_(CN)_5_. In particular, KCd[Cu(CN)_2_]_3_ is a new melting analog in the well‐established family of 3D wine‐rack‐type CN^−^‐bridged structures. We have demonstrated that both crystal‐interface interactions and the presence of low‐coordinate Cu nodes exert substantial effects on the phase transition. Accordingly, this study introduces a new design paradigm for constructing meltable coordination frameworks, emphasizing the tuning of crystalline surfaces and the use of low‐coordinate metal nodes. It is noteworthy that while [Cu(CN)_2_]^−^ appears to be a straightforward building unit, its use in the synthesis of coordination frameworks has not been previously reported. Therefore, this study shows that the synthesis of novel compounds through the conversion of hydrated parents into distinct crystalline phases offers a simple and promising avenue for obtaining unexpected materials, often unattainable via conventional synthetic routes. Although the dehydration process can entail complex intracrystalline reactions, frequently yielding mixed‐phase products, careful characterization of these compounds and comprehensive investigation of their physical properties through techniques such as electron microscopy and various spectroscopies can reveal new materials, thereby advancing the field of materials chemistry.

## Supporting Information

The Supporting Information is available free of charge at Experimental Section, crystal parameters, VT‐PXRD patterns, VT‐IR spectra, TG‐DTA curves, TEM‐EDX results, microscopic images, DSC curves, and MD simulation results

## Accession code

MicroED raw images have been deposited to XRDa (accession code XRD‐283). Refined coordinates of compounds have been deposited in CCDC 2414728 (**1**), 2414729 (**2**), and 2414730 (**3**) and COD 5000584 (**2**) and 5000585 (**3**). Scripts for MicroED data collection and processing are available at https://github.com/GKLabIPR/MicroED.

## Conflict of Interest

The authors declare no conflict of interest.

## Supporting information



Supporting Information

Supporting Information

Supporting Information

Supporting Information

Supporting Information

## Data Availability

The data that support the findings of this study are available from the corresponding authors upon reasonable request.
