# Supplementary material for: Reversible and Massive Structural Transformation in Meltable Cyanido‐bridged Coordination Polymer Crystals
Source: Chemistry. 2025 Oct 25;31(66):e02640. doi: 10.1002/chem.202502640 (PMC12648463; doi:10.1002/chem.202502640)
Supplement: Supplementary file 1 — Supporting Information [file CHEM-31-e02640-s004.docx]

Synthesis

Cd(CN)_2_

A 7-M KCN solution was slowly added to 15 mL of an aqueous solution of Cd(NO_3_)_2_∙4H_2_O (10 g, 32.37 mmol) until a white precipitate formed. Cd(CN)_2_ was obtained as a white powder after filtration followed by vigorous washing with H_2_O and drying. Anal. Found (calcd.) for CdC_2_N_2_H_0.6_O_0.3_ (169.8): C, 14.11 (14.13); H, 0.07 (0.35); N, 16.23 (16.49).

K_2_Cd(CN)_4_

Cd(CN)_2_ (600 mg, 3.65 mmol) was suspended in an aqueous solution of KCN (474 mg, 7.31 mmol) 60 mL followed by stirring overnight. After confirming the complete dissolution, a white precipitation of K_2_Cd(CN)_4_ was produced. Anal. Found (calcd.) for K_2_CdC_4_N_4_HO_0.5_ (303.4): C, 15.87 (15.82); H, 0.04 (0.33); N, 18.28 (18.46).

K_2_Cd(H_2_O)Cu_4_(CN)_8_∙1.5H_2_O (**1**)

CuCN (200 mg, 0.57 mmol) was added to an aqueous solution of K_2_Cd(CN)_4_ (320mg, 1.09 mmol) and K[Ntf_2_] (80 mg, 0.25 mmol) 30 mL. The suspension was vigorously stirred while heating at 363 K. After cooling to room temperature, the precipitate was removed via filtration. Colorless block crystals of **1** were obtained after standing for several days. Note that colorless crystals of other compounds such as **3**∙*n*H_2_O and Cd(H_2_O)_2_Cu(CN)_3_∙2H_2_O also appeared. Thus, we carefully separated these crystals by their shapes and powder X-ray diffraction (PXRD) results measured after grinding. Anal. Found (calcd.) for K_2_CdCu_4_C_8_N_8_H_5_O_2.5_ (697.6): C, 13.73 (13.76); H, 0.66 (0.72); N, 15.87 (16.06).

Composite of KCd[Cu(CN)_2_]_3_ (**2**) and K_2_Cu_3_(CN)_5_ (**3**)

The beige powders of the composite containing **2** and **3** were obtained by thermal treatment of **1** at 474 K overnight.

K_2_Cu_3_CN_5_ (**3**)^1^

This compound was synthesized with the reported method after slight modificaion. KCN (65 mg, 1.0 mmol), K_3_Fe(CN)_6_ (50 mg, 0.15 mmol), and CuCN (90 mg, 1.0 mmol) were mixed and carefully glinded. The mixed powder was added into 20 mL MeCN, then heated at 413 K for 2 days. After cooling to room temperature, we obtained blown powder of **3** with residual orange single crystals of K_3_Fe(CN)_6_. Note that **3** and K_3_Fe(CN)_6_ were separated as much as possible.

Rehydrated mixture sample of **2** and **3** (**CdCu_rehyd_**)

The beige powders of **2** and **3** (with some residue) were exposed to H_2_O vapor, yielding the powder sample **CdCu_rehyd_**. Anal. Found (calcd.) for K_2_CdCu_4_C_8_N_8_H_6_O_3_ (706.6): C, 13.54 (13.59); H, 0.73 (0.85); N, 15.57 (15.85).

Physical measurements

The single-crystal X-ray data of **1** were recorded using a Bruker D8 Venture diffractometer equipped with a PHOTON II detector with Mo Kα radiation (λ = 0.71073 Å). Data integration and reduction were performed using APEX4 software. The structures were solved using Olex2^2^ and the ShelXT^3^ structure solution program using direct methods and were refined using the ShelXL^4^ refinement package with least-squares minimization. The temperature was controlled by N_2_ gas flow using an Oxford Cryosystems 800 Series Cobra controller. Hydrogen atoms were included in idealized positions and refined using a riding model. Variable-temperature PXRD (VT-PXRD) was performed at 100 K–500 K using the SAGA-LS, BL-15 (λ = 1.08 Å) in the 2θ range of 2.00°−56.465° with a step width of 0.02° at each temperature. The powder samples with homogeneous granularity were sealed in a glass capillary with an internal diameter of 0.4 mm. The sample temperatures were controlled by dry dinitrogen flow using a Rigaku GN_2_ apparatus. VT-PXRD data were collected at 300 K–580 K using a Miniflex 600-C (Rigaku) with a D/teX Ultra II detector using CuK α radiation (λ = 1.54184 Å) and the temperature-control device BTS 500 (Anton Paar) with N_2_ flow. The lattice constants were calculated using SmartLab Studio II (Rigaku). Infrared (IR) spectral measurements in air were performed using a PerkinElmer Spectrum Two FTIR spectrometer (Perkin Elmer) equipped with an ATR accessory. VT-IR spectra were recorded using Spectrum Two with Spotlight 200i (Perkinelmer). Sample temperature was controlled by TC-33A controller (Daico). Thermogravimetric analysis (TGA) was performed at 3-K min^−1^ using Rigaku Instrument Thermo plus TG 8120 in. a N_2_ atmosphere with a Pt and Al_2_O_3_ pan. Scanning electron microscopy images were obtained using the desktop scanning electron microscope Phenom Pro (Thermo Fisher Scientific). The crystals were affixed onto electrically conductive carbon tape, followed by Au sputtering to enhance electrical conductivity. Differential scanning calorimetry (DSC) analyses were performed using a HITACHI DSC 7020 under nitrogen flow. The adsorption isotherms were collected using BELSORP-MAX volumetric adsorption equipment (Microtrac BEL Corp.). The samples were dehydrated by heating at 473 K for 12 h before the measurements.

VT-PXRD measurements for CdCu_dehyd_ were performed at the SPring-8 BL02B2 beamline equipped with MYTHEN2 solid-state detectors^5,6^ using an incident beam monochromatized to λ = 0.799585 Å. The well-ground powder sample was loaded into a borosilicate capillary (0.4 mm in diameter). The capillary was rotated during the measurements for improved averaging of the powder pattern data. The sample temperature was controlled by nitrogen gas flow.
Three-dimensional (3D) electron diffraction (MicroED) data collection and analysis were performed following our established protocol^7^ that involved semiautomated data collection by SerialEM^8^ and multicrystal analysis in DIALS^9,10^. Data processing was parallelized with GNU parallel^11^. **CdCu_dehyd_** powders on a QuantiFoil Mo R0.6/1.0 grid were loaded onto a Talos Arctica electron microscope. The microscope was operated at an accelerating voltage of 200 kV. The parallel illumination had a diameter of ~1.6 um and an electron flux of ~0.06 electron/Å2/s. The virtual camera distance was set to 615.5 mm. More than 950 locations picked from the SerialEM montage images (Fig. S4) were screened without rotation, and 309 datasets were collected while the grid was rotated at ~1.248°/s. Diffraction images were recorded on a Falcon 3 direct electron detector^12^ in the integration mode at ~0.435°/fraction. The sample was kept at ~78 K during measurement.

Initial processing in *P*1 revealed at least three clusters (Fig. S5). The images were reprocessed with Bravais lattice constraints and prior cell information corresponding to each cluster. High-resolution isomorphic subsets were selected by xia2.multiplex^13^ and scaled with dials.scale^14^ (Tables S3 and S4). Two clusters were identified as KCd[Cu(CN)_2_]_3_ (**2**, 35 merged crystals out of 85 indexed crystals) and K_2_Cu_3_(CN)_5_ (**3**, 23 crystals out of 86 indexed crystals), whereas the third cluster could not be phased. Note that the ratio of **2** and **3** does not reflect the composition of the bulk material because only particles of suitable sizes can be measured by MicroED. In addition, only the most dominant component was indexed when multiple components were simultaneously irradiated by the beam. The merged intensities were phased by SHELXD^15^, and the structures were refined kinematically by SHELXL^4^ in the Olex2^2^ GUI. Because the dynamical effects were ignored, the refinement statistics were worse than those typically found via X-ray crystallography.

Transmission electron microscopy (TEM)–energy-dispersive X-ray spectroscopy measurements were performed using a ThemisZ transmission electron microscope (Thermo Fisher Scientific), which was operated in scanning (scanning transmission electron microscopy) mode at 300 kV, with a probe size of ~0.2 nm (full width at half maximum) and a probe current of ~200 pA.

TEM samples were prepared by dispersing the powder in chloroform and, following sonication, pipetting a few drops of the solution onto Mo TEM grids covered with a holey carbon film. The following lines were used for mapping and quantification: C-K, N-K, O-K, K-K, Cd-L, and Cu-K.

Computation

The crystal structures of KCd[Cu(CN)_2_]_3_, KCd[Ag(CN)_2_]_3_, and KCd[Au(CN)_2_]_3_ were optimized using the Vienna Ab initio Simulation Package^16,17^. The atomic positions and cell parameters were fully optimized until the forces on all atoms were less than 0.05 eV/Å. The generalized gradient approximation was used, as described by Perdew, Burke, and Ernzerhof^18^. The Kohn–Sham equations were solved using a plane-wave basis set with the projector-augmented wave method^19^ and a cutoff energy of 400 eV. The convergence threshold for the self-consistent field iteration was set to 1.0 × 10^–4^ eV. The Γ-centered *k*-point meshes with a *k* spacing of 2π × 0.05 Å^–1^ were employed to sample the Brillouin zone. Grimme’s D3 dispersion correction with Becke–Johnson damping was applied^20^. The crystal structures were optimized based on the experimentally obtained crystal structures. However, in the experimentally determined structures, the occupancy of the K atom sites was 0.5. Therefore, we created a 2 × 2 × 2 supercell and randomly generated 10 crystal structures for each compound under the condition that each K site had a 50% probability of being occupied by a K atom. These structures were optimized, and the most stable crystal structure was selected for subsequent analyses.

To calculate the formation enthalpies of KCd[Cu(CN)_2_]_3_, KCd[Ag(CN)_2_]_3_, and KCd[Au(CN)_2_]_3_, we optimized the crystal structures of the elemental reference phases: body-centered cubic K, hexagonal close-packed Cd, face-centered cubic Cu, Ag, and Au, and graphite. The structure of the gas-phase N_2_ molecule was also optimized, and their respective energies were obtained.

To understand the dynamic behavior of KCd[Cu(CN)_2_]_3_, KCd[Ag(CN)_2_]_3_, and KCd[Au(CN)_2_]_3_ at high temperatures, first-principles molecular dynamics simulations were performed using the optimized 2 × 2 × 2 supercell structures of these compounds as the initial structures. The simulations were performed in an NVT ensemble using a Nosé–Hoover thermostat^21^. The temperature was set to 1,000 K, the time step was set to 1 fs, and the total simulation time was set to 1,500 fs. Only Γ-point sampling was used for the first Brillouin zone. The other calculation settings were identical to those described above. The radial distribution functions were calculated for the trajectories in the time intervals of 0–500, 500–1,000, and 1,000–1,500 fs.

Table S1 Crystal parameters of **1**

| **Temp. (K)** | **100** |
| --- | --- |
| **CCDC number** | 2414728 |
| **Crystal System** | Monoclinic |
| **Space Group** | *C*2/*c* |
| **Formula** | K_2_CdCu_4_C_7_N_9_H_0.5_O_2.5_ |
| ***a* (Å)** | 19.370(5) |
| ***b* (Å)** | 15.819(4) |
| ***c* (Å)** | 12.640(3) |
| ***β* (˚)** | 109.423(6) |
| ***V* (Å^3^)** | 3652.65 |
| **Z** | 8 |
| **R1** | 2.26 |
| **Rwp** | 5.99 |
| **G.O.F** | 1.137 |


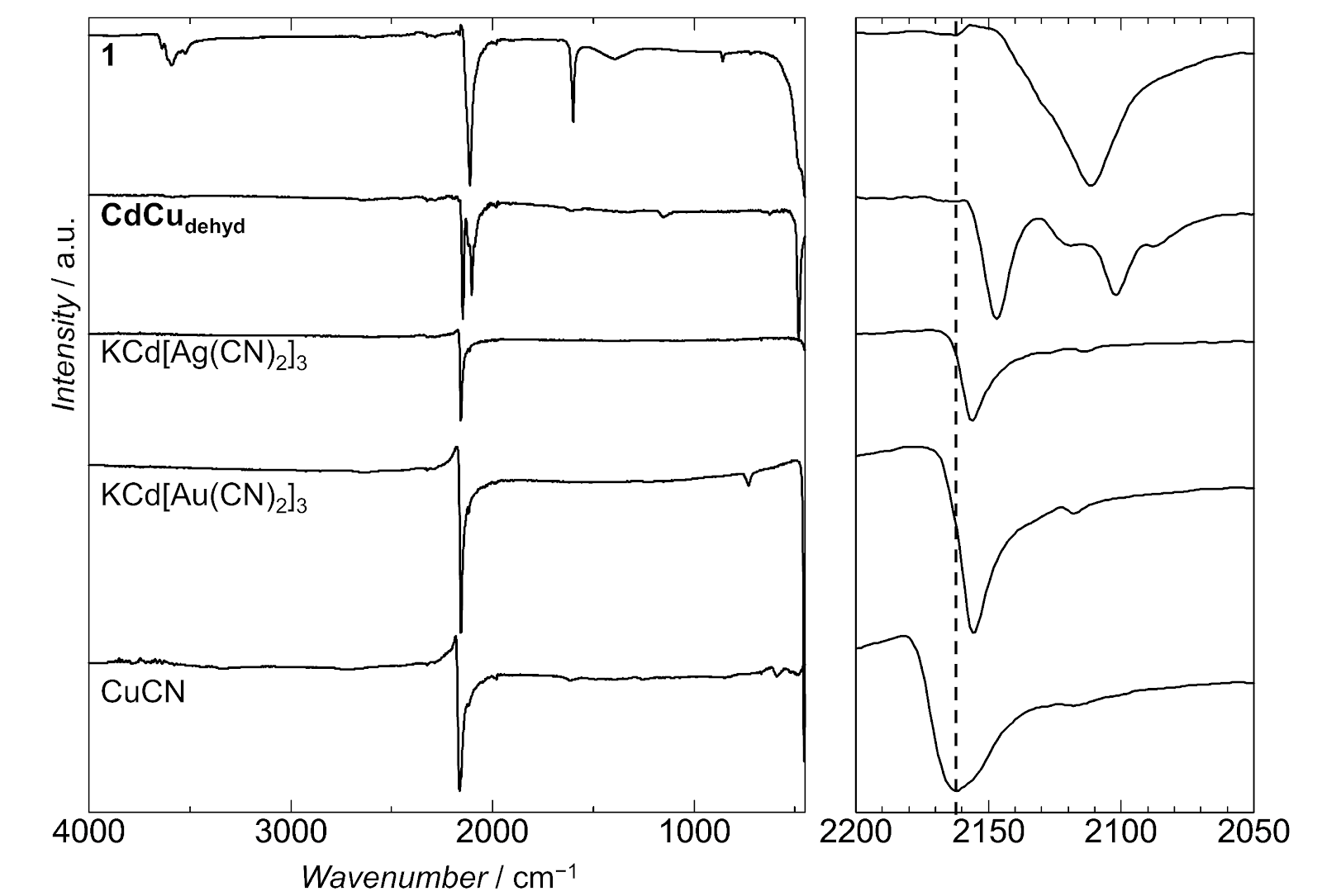
 Fig. S1 IR spectra of **1**, **CdCu_dehyd_**, KCd[Ag(CN)_2_]_3_, KCd[Au(CN)_2_]_3,_ and CuCN


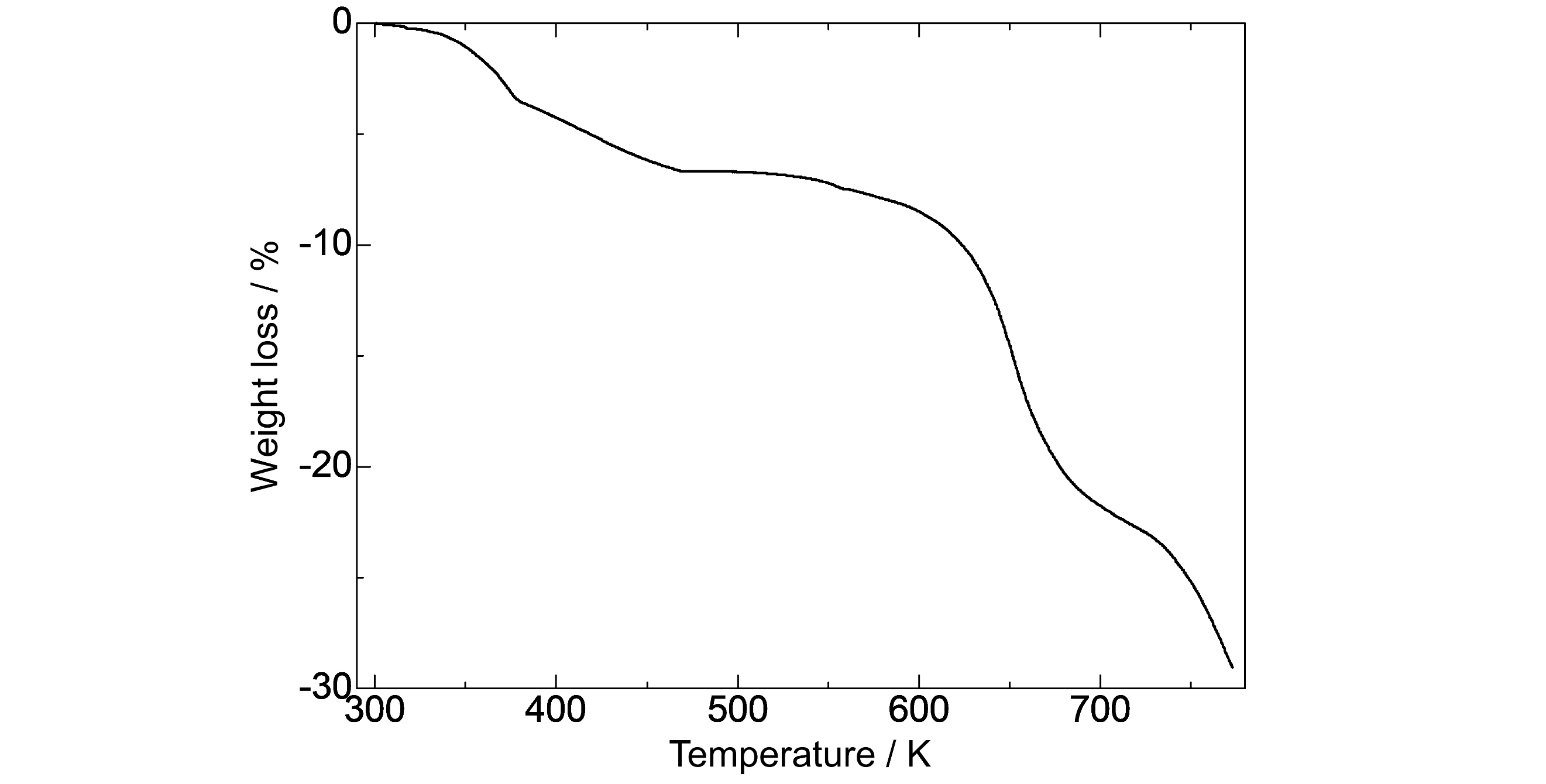


Fig. S2 TGA result of **1**.


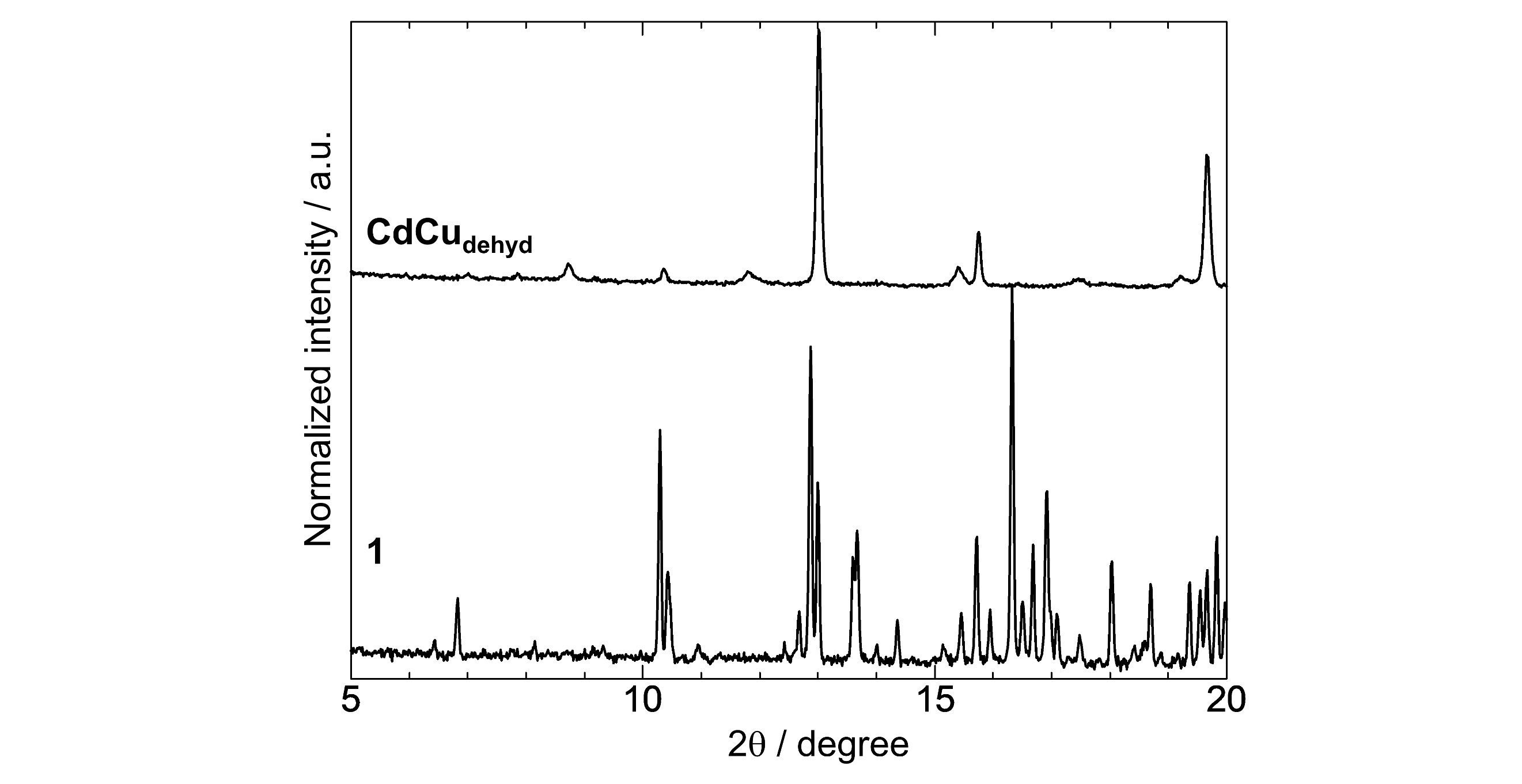


Fig. S3 PXRD patterns of **CdCu_dehyd_** and **1**. These data were recorded on λ = 1.08Å.

Table S2 Crystal parameters of KCd[Cu(CN)_2_]_3_ (**2**) and K_2_Cu_3_(CN)_5_ (**3**)

| **Compound** | **KCd[Cu(CN)_2_]_3_ (2)** | **K_2_Cu_3_(CN)_5_ (3)** |
| --- | --- | --- |
| **Temp. (K)** | 79 | 79 |
| **CCDC number** | 2414729 | 2414730 |
| **COD number** | 5000584 | 5000585 |
| **Crystal System** | Hexagonal | Monoclinic |
| **Space Group** | *P*-31*m* | *C*2/*c* |
| **Formula** | K_2_CdCu_3_C_6_N_6_ | K2Cu3C5N5 |
| ***a* (Å)** | 6.778 | 17.355 |
| ***b* (Å)** | 6.778 | 7.951 |
| ***c* (Å)** | 8.005 | 7.695 |
| ***β* (˚)** | 90 | 1061.82 |
| ***V* (Å^3^)** | 318.489 | 16.54 |
| **Z** | 1 | 4 |
| **R1** | 23.33 | 16.54 |
| **Rwp** | 55.38 | 38.61 |
| **G.O.F** | 3.472 | 1.285 |

Table S3 Merging statistics of KCd[Cu(CN)_2_]_3_ (**2**)

| **d_max** | **d_min** | **#obs** | **#uniq** | **mult.** | **%comp** | **<I/sI>** | **r_pim** | **cc1/2** |
| --- | --- | --- | --- | --- | --- | --- | --- | --- |
| 5.87 | 1.48 | 1,308 | 47 | 27.83 | 100 | 38.6 | 0.033 | 0.996* |
| 1.48 | 1.18 | 1,277 | 37 | 34.51 | 100 | 23.7 | 0.051 | 0.988* |
| 1.18 | 1.03 | 1,508 | 44 | 34.27 | 100 | 13.4 | 0.067 | 0.918* |
| 1.03 | 0.94 | 1,332 | 36 | 37.00 | 100 | 11.7 | 0.071 | 0.924* |
| 0.94 | 0.87 | 1,414 | 36 | 39.28 | 100 | 7.6 | 0.075 | 0.928* |
| 0.87 | 0.82 | 1,505 | 41 | 36.71 | 100 | 6.1 | 0.095 | 0.952* |
| 0.82 | 0.78 | 1,481 | 40 | 37.02 | 100 | 3.9 | 0.122 | 0.913* |
| 0.78 | 0.74 | 1,083 | 28 | 38.68 | 100 | 3.2 | 0.119 | 0.774* |
| 0.74 | 0.72 | 1,669 | 42 | 39.74 | 100 | 3.0 | 0.118 | 0.853* |
| 0.72 | 0.69 | 1,575 | 38 | 41.45 | 100 | 2.3 | 0.143 | 0.634* |
| 0.69 | 0.67 | 1,247 | 34 | 36.68 | 100 | 1.3 | 0.200 | 0.730* |
| 0.67 | 0.65 | 1,591 | 42 | 37.88 | 100 | 1.2 | 0.220 | 0.549* |
| 5.87 | 0.65 | 16,990 | 465 | 36.54 | 100 | 10.3 | 0.060 | 0.995* |

Table S4 Crystallographic merging statistics of K_2_Cu_3_(CN)_5_ (**3**)

| **d_max** | **d_min** | **#obs** | **#uniq** | **mult.** | **%comp** | **<I/sI>** | **r_pim** | **cc1/2** |
| --- | --- | --- | --- | --- | --- | --- | --- | --- |
| 5.27 | 1.59 | 3,201 | 153 | 20.92 | 100 | 35.8 | 0.043 | 0.987* |
| 1.59 | 1.27 | 3,771 | 149 | 25.31 | 100 | 35.5 | 0.042 | 0.987* |
| 1.27 | 1.11 | 3,677 | 142 | 25.89 | 100 | 28.3 | 0.053 | 0.990* |
| 1.11 | 1.01 | 3,960 | 149 | 26.58 | 100 | 24.6 | 0.058 | 0.984* |
| 1.01 | 0.94 | 3,876 | 144 | 26.92 | 100 | 22.7 | 0.061 | 0.975* |
| 0.94 | 0.88 | 3,713 | 137 | 27.10 | 100 | 21.6 | 0.064 | 0.963* |
| 0.88 | 0.84 | 4,185 | 154 | 27.18 | 100 | 17.7 | 0.072 | 0.972* |
| 0.84 | 0.80 | 3,896 | 137 | 28.44 | 100 | 15.2 | 0.079 | 0.949* |
| 0.80 | 0.77 | 4,047 | 144 | 28.10 | 100 | 15.0 | 0.080 | 0.942* |
| 0.77 | 0.74 | 4,005 | 143 | 28.01 | 100 | 11.9 | 0.095 | 0.915* |
| 0.74 | 0.72 | 3,680 | 132 | 27.88 | 100 | 10.7 | 0.098 | 0.889* |
| 0.72 | 0.70 | 4,089 | 142 | 28.80 | 100 | 9.8 | 0.105 | 0.885* |
| 0.70 | 0.68 | 4,166 | 139 | 29.97 | 100 | 9.2 | 0.111 | 0.749* |
| 0.68 | 0.66 | 4,074 | 153 | 26.63 | 100 | 6.9 | 0.134 | 0.781* |
| 0.66 | 0.65 | 4,031 | 132 | 30.54 | 100 | 7.0 | 0.140 | 0.693* |
| 0.65 | 0.64 | 3,866 | 138 | 28.01 | 100 | 5.6 | 0.158 | 0.682* |
| 0.64 | 0.62 | 4,097 | 143 | 28.65 | 100 | 5.2 | 0.163 | 0.798* |
| 0.62 | 0.61 | 4,046 | 141 | 28.70 | 100 | 4.6 | 0.191 | 0.686* |
| 0.61 | 0.60 | 4,642 | 157 | 29.57 | 100 | 3.9 | 0.224 | 0.636* |
| 0.60 | 0.59 | 3,428 | 121 | 28.33 | 100 | 3.4 | 0.239 | 0.661* |
| 5.27 | 0.59 | 78,450 | 2,850 | 27.53 | 100 | 14.9 | 0.061 | 0.985* |


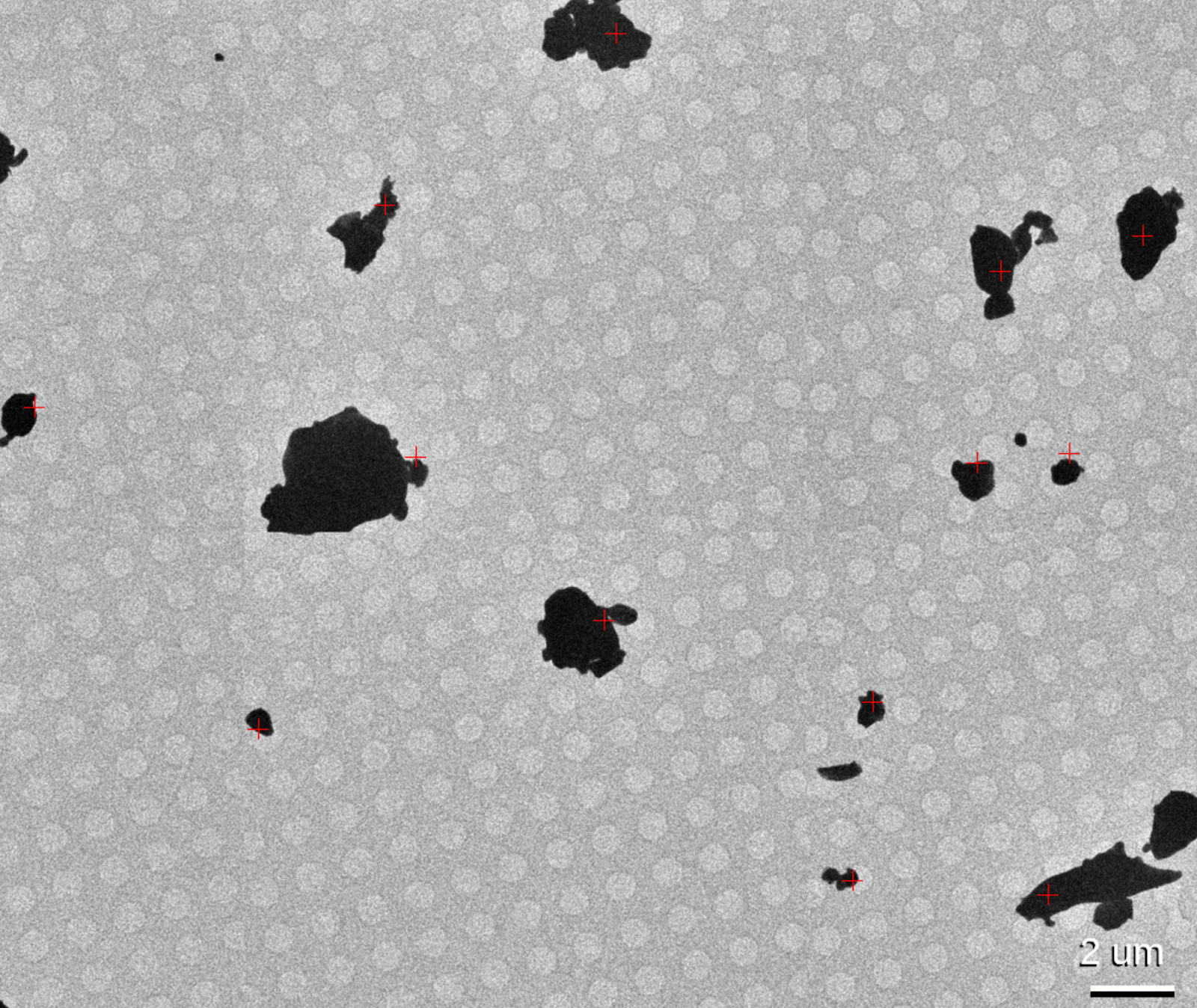


Fig. S4 A real-space image of MicroED grid of **CdCu_dehyd_**. Image discontinuities are caused by errors in montage alignment by SerialEM. The red crosses indicate the positions of data collection.


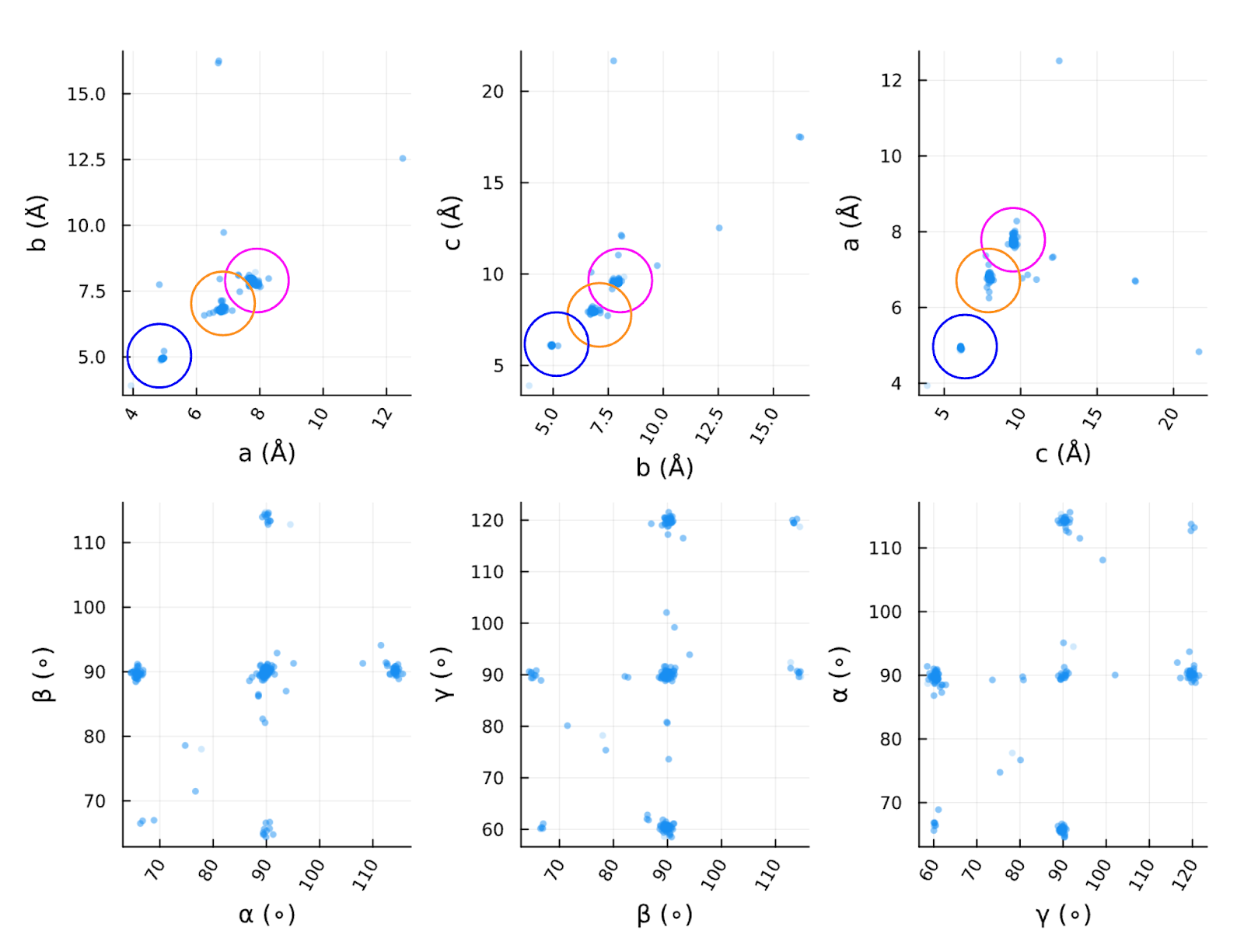


Fig. S5 Scatter plot of unit cell parameters obtained by MicroED. 109 indexed crystals better than 1.2 Å (of 309 measured and more than 950 screened positions) are shown. This is a raw distribution without applying reindexing and Bravais lattice constraints and includes misindexed and/or weakly diffracting crystals rejected in subsequent processing steps.
The pink cluster can be reindexed to *mC* and corresponds to K_2_Cu_3_(CN)_5_ (**3**). The orange cluster corresponds to KCd[Cu(CN)_2_]_3_ (**2**). The identity of the blue cluster remains unknown, but the PXRD of **CdCu_dehyd_** can be interpreted as the sum of the simulated PXRD patterns of **2** and **3**, indicating that the amount of a compound suggested by the blue cluster is very small.


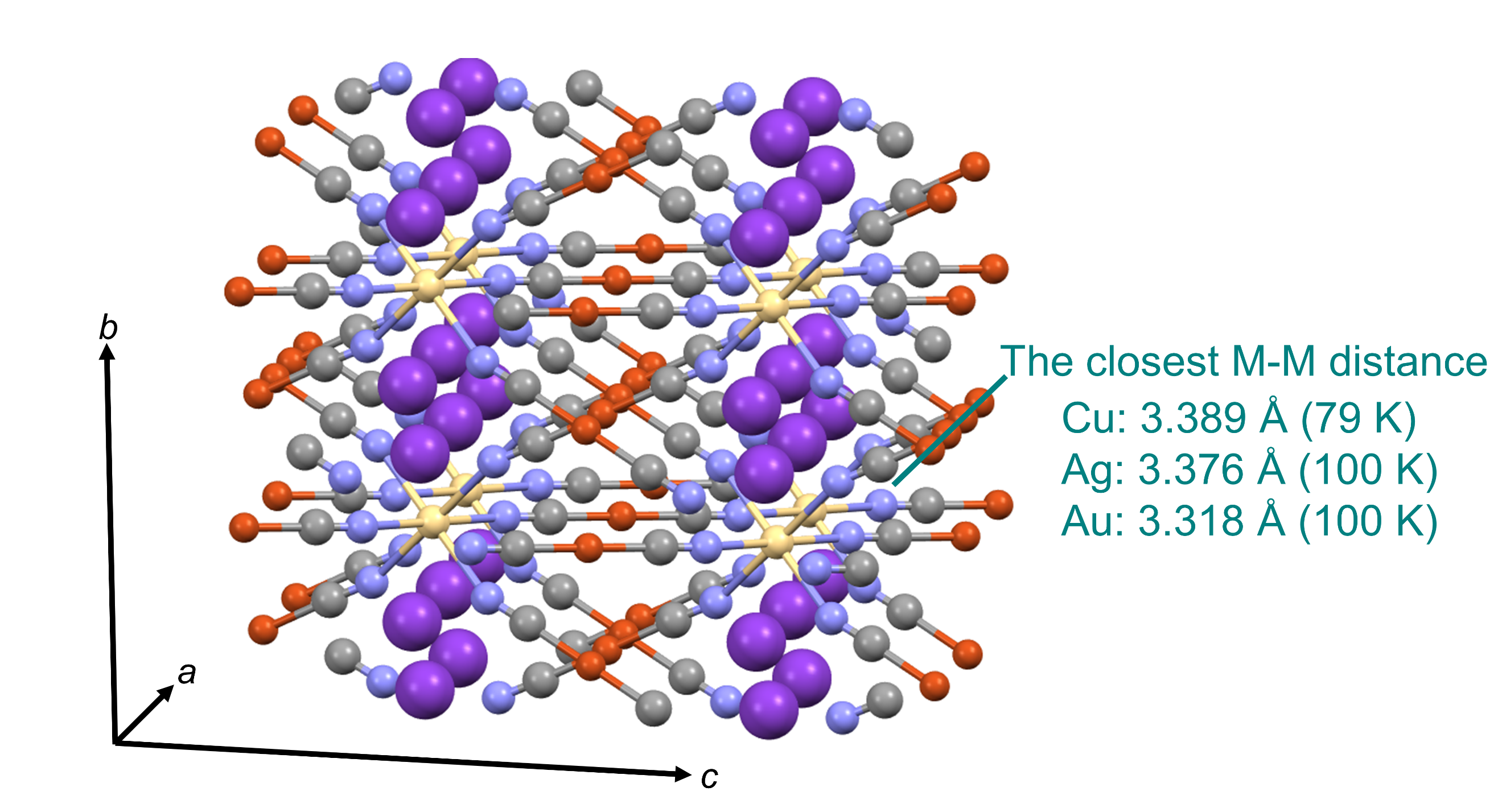


Fig. S6 The closest distances between the metal centers of [M(CN)_2_]^−^ units along the *a*-axis in KCd[M(CN)_2_]_3_ (M = Cu, Ag, and Au)


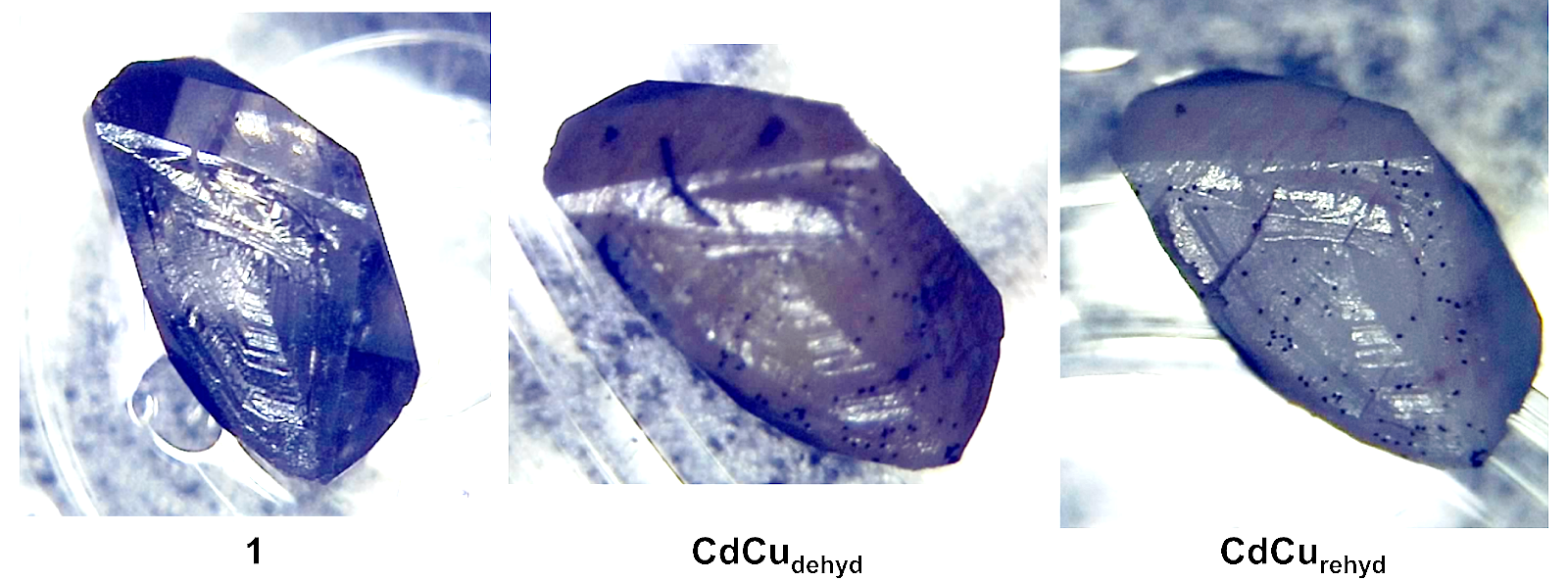


Fig. S7 Single-crystal pictures of **1**, **CdCu_dehyd_**, and **CdCu_rehyd_**


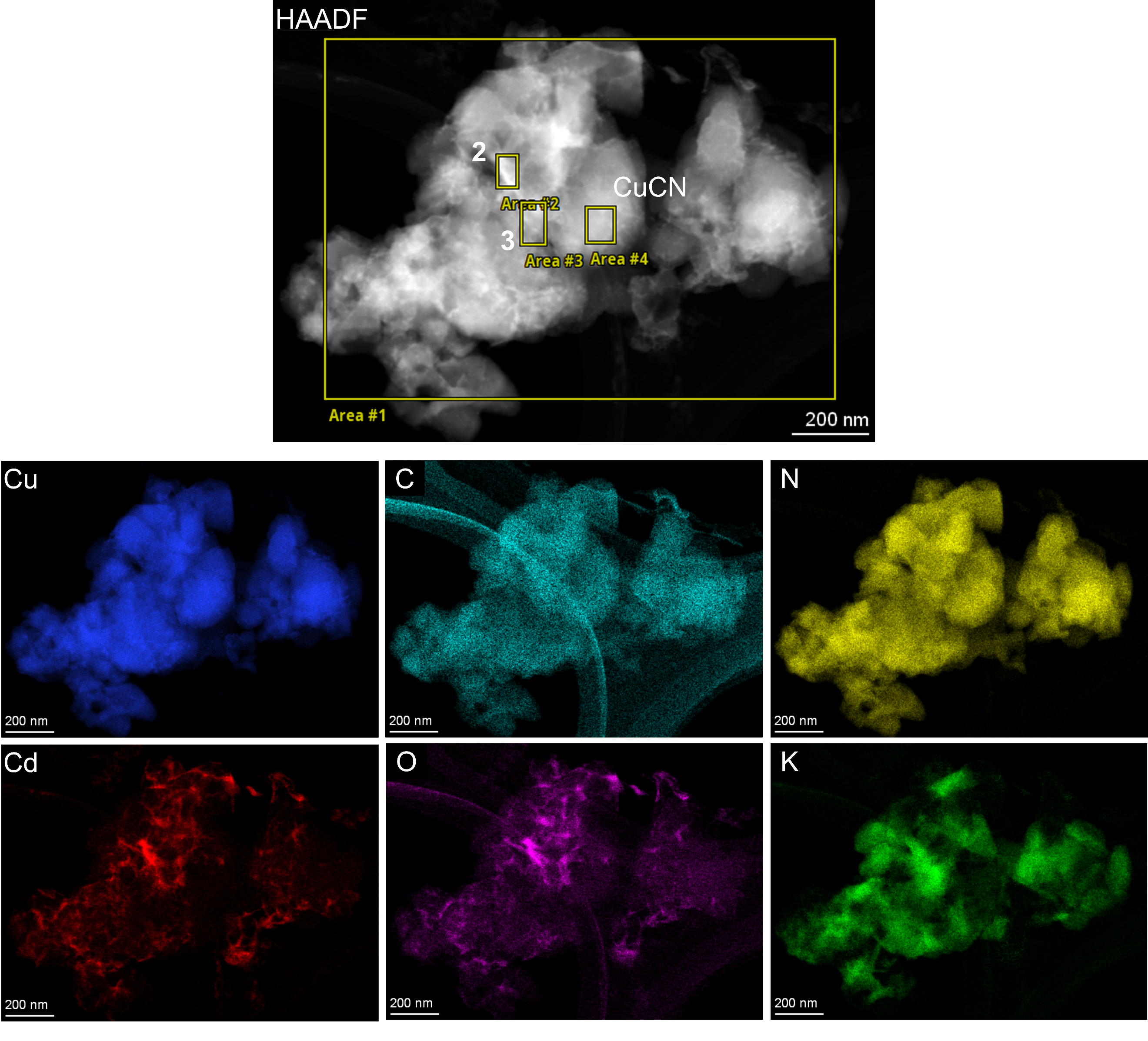


Fig. S8 TEM image and Elemental mapping of **CdCu_dehyd_**

Table S5 Elemental ratio (atomic %) of Areas 1, 2, 3, and 4 in Fig. S7

| **Element** | **Area 1** | **Area 2** | **Area 3** | **Area 4** |
| --- | --- | --- | --- | --- |
| **C** | 25.42 ± 1.97 | 24.84 ± 1.64 | 22.07 ± 1.66 | 20.74 ± 1.87 |
| **N** | 28.31 ± 4.33 | 22.88 ± 3.68 | 25.69 ± 4.06 | 31.36 ± 4.76 |
| **O** | 3.65 ± 0.76 | 11.50 ± 2.16 | 3.71 ± 0.77 | 1.51 ± 0.33 |
| **K** | 4.92 ± 0.91 | 9.41 ± 1.61 | 12.34 ± 2.10 | 1.33 ± 0.26 |
| **Cu** | 35.94 ± 3.90 | 24.31 ± 2.95 | 33.61 ± 3.72 | 44.90 ± 4.54 |
| **Cd** | 1.77 ± 0.24 | 7.06 ± 0.89 | 2.58 ± 0.35 | 0.17 ± 0.02 |
| **Assumed formula** |  | KCd[Cu(CN)_2_]_3_ | K_2_Cu_3_(CN)_5_ | CuCN |


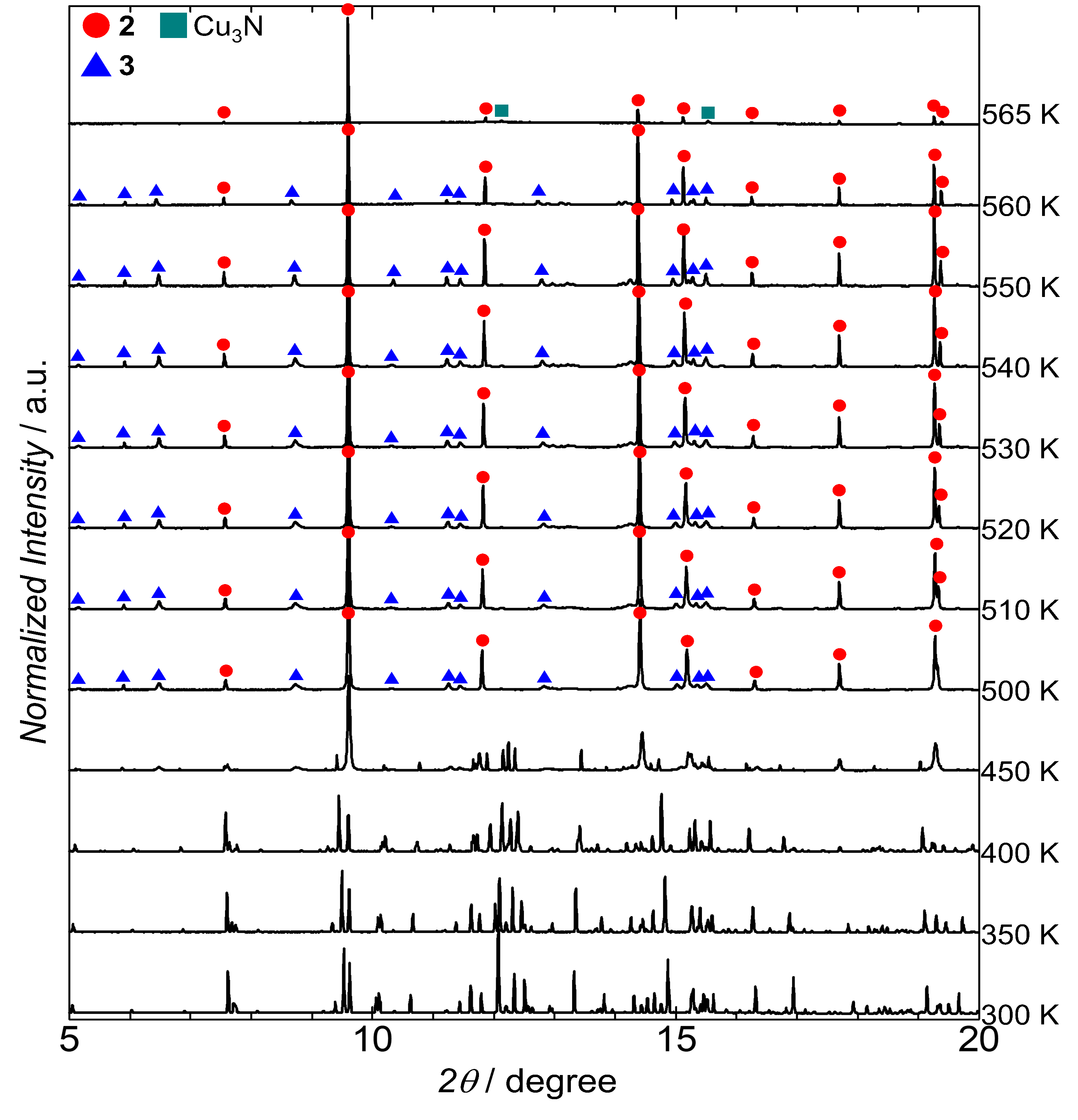


Fig. S9 VT-PXRD patterns of **1** in the heating process at 300 K–565 K under vacuum (λ = 0.79985 Å, 10 K/min). The red circles, blue triangles and, green squares indicate the diffraction peaks of **2**, **3**, and Cu_3_N, respectively.





Fig. S10 Time-dependent PXRD results under vacuum at 565 K (λ = 0.799585 Å). The red circles and green squares indicate the diffraction peaks of **2** and Cu_3_N, respectively.


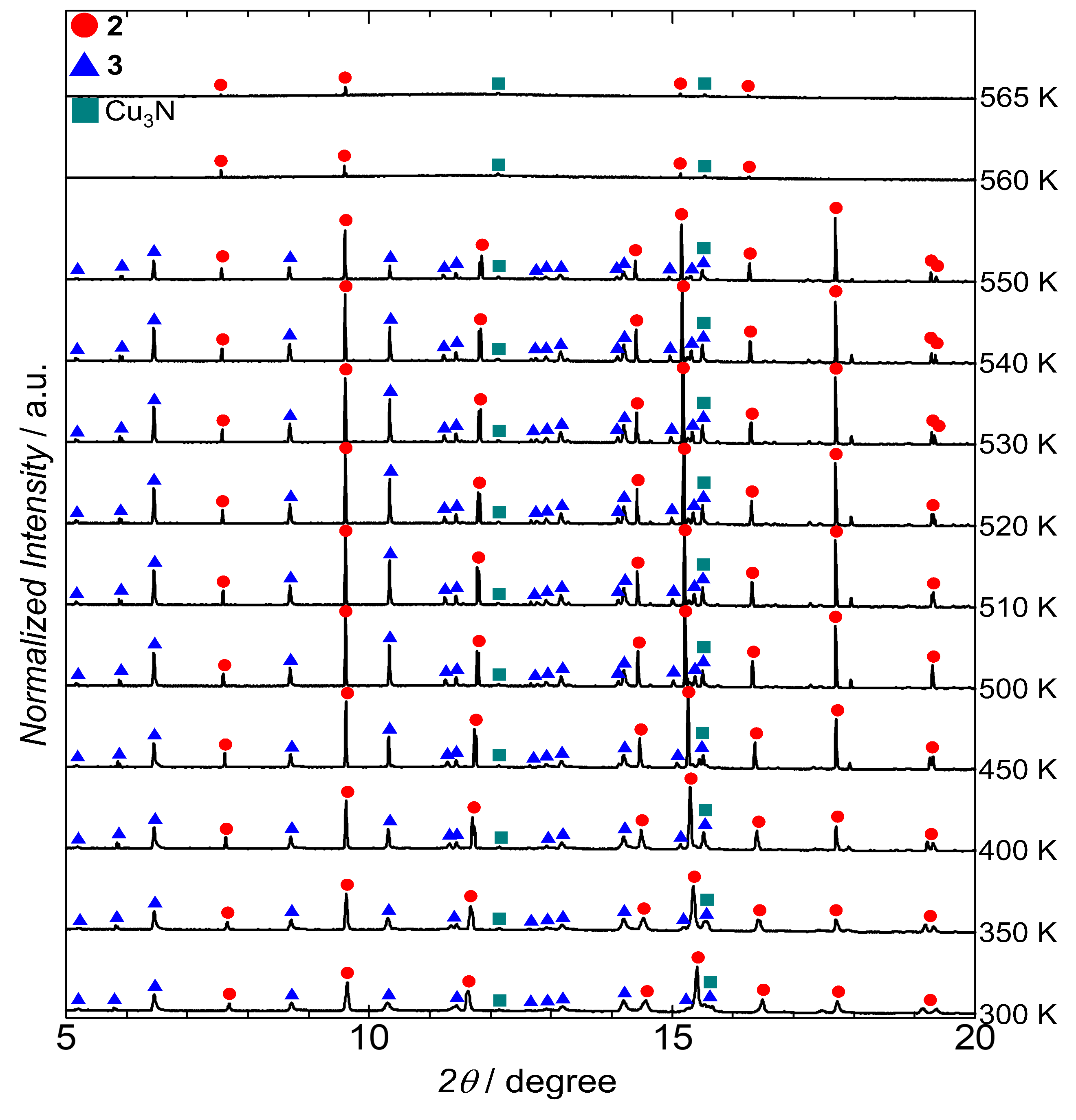


Fig. S11 VT-PXRD patterns in the cooling process from 565 K to 300 K (λ = 0.79985 Å, 10 K/min). The red circles, blue triangles, and green squares indicate the diffraction peaks of **2**, **3**, and Cu_3_N, respectively.


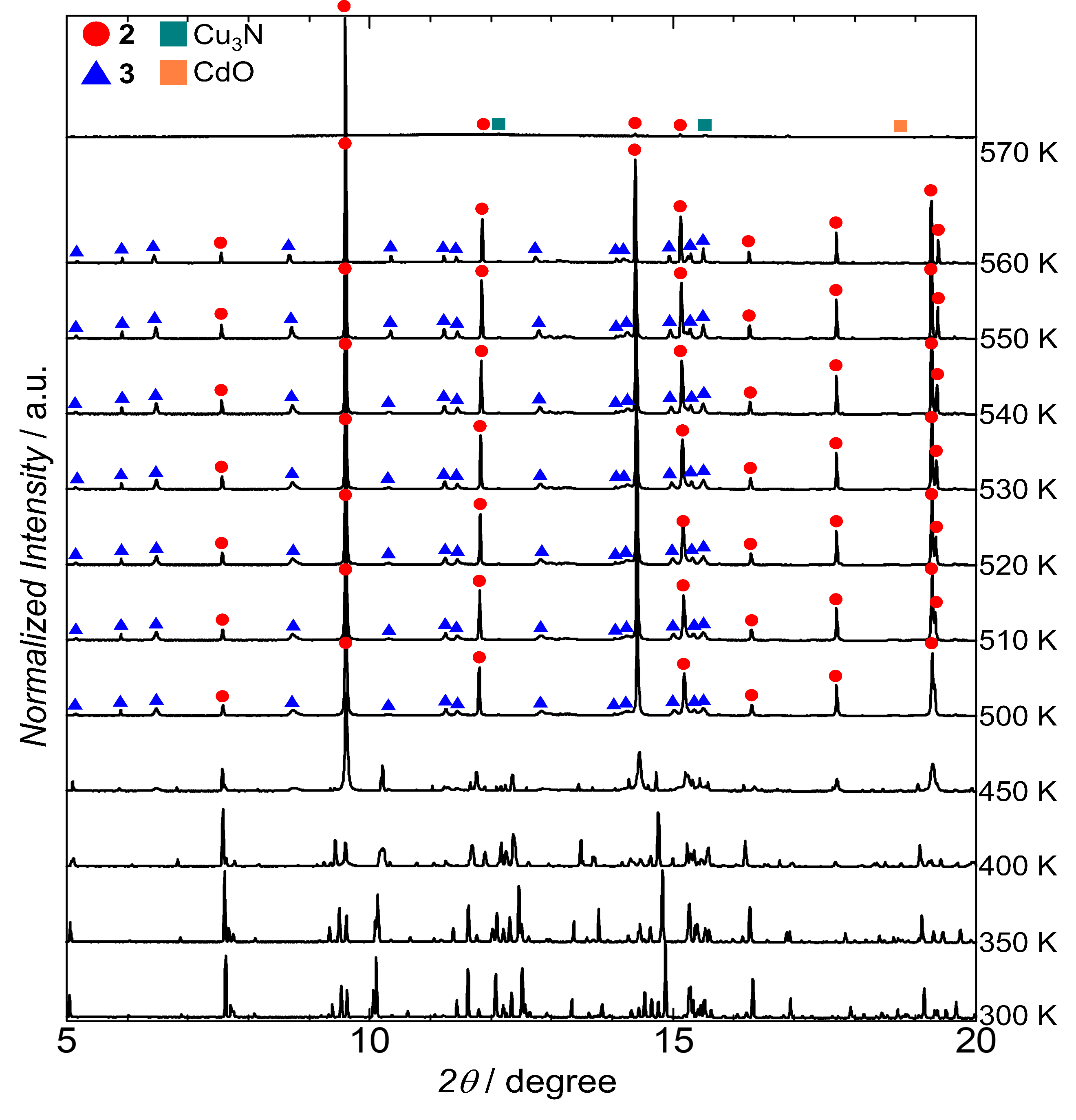


Fig. S12 VT-PXRD patterns of **1** in the heating process at 300 K–570 K under vacuum (λ = 0.79985 Å, 10 K/min). The red circles, blue triangles, green squares, and orange squares indicate the diffraction peaks of **2**, **3**, Cu_3_N, and CdO, respectively.





Fig. S13 Time-dependent PXRD results under vacuum at 570 K (λ = 0.799585 Å). The red circles, green squares, and orange squares indicate the diffraction peaks of **2**, Cu_3_N, and CdO, respectively.


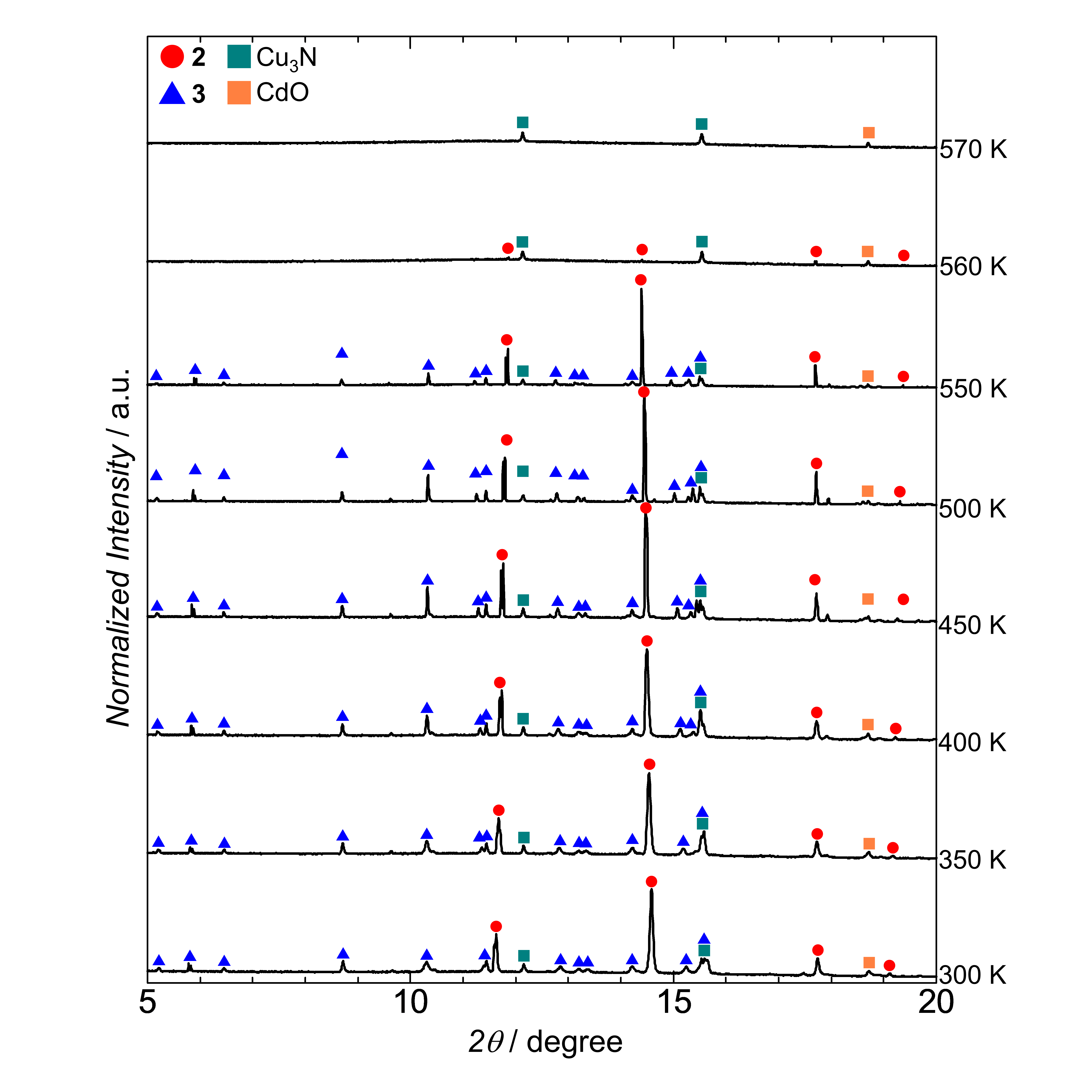


Fig. S14 VT-PXRD patterns in the cooling process from 570 K to 300 K (λ = 0.79985 Å, 10 K/min). The red circles, blue triangles, green squares, and orange squares indicate the diffraction peaks of **2**, **3**, Cu_3_N, and CdO, respectively.





Fig. S15 VT-PXRD patterns of **1** in the heating process at 300 K–560 K under vacuum (λ = 0.79985 Å, 10 K/min). The red circles and blue triangles indicate the diffraction peaks of **2** and **3**, respectively





Fig. S16 Time-dependent PXRD results under vacuum at 560 K (λ = 0.799585 Å). The red circles and blue triangles indicate the diffraction peaks of **2** and **3**, respectively





Fig. S17 VT-PXRD patterns in the cooling process from 560 K to 300 K (λ = 0.79985 Å, 10 K/min). The red circles and blue triangles indicate the diffraction peaks of **2** and **3**, respectively


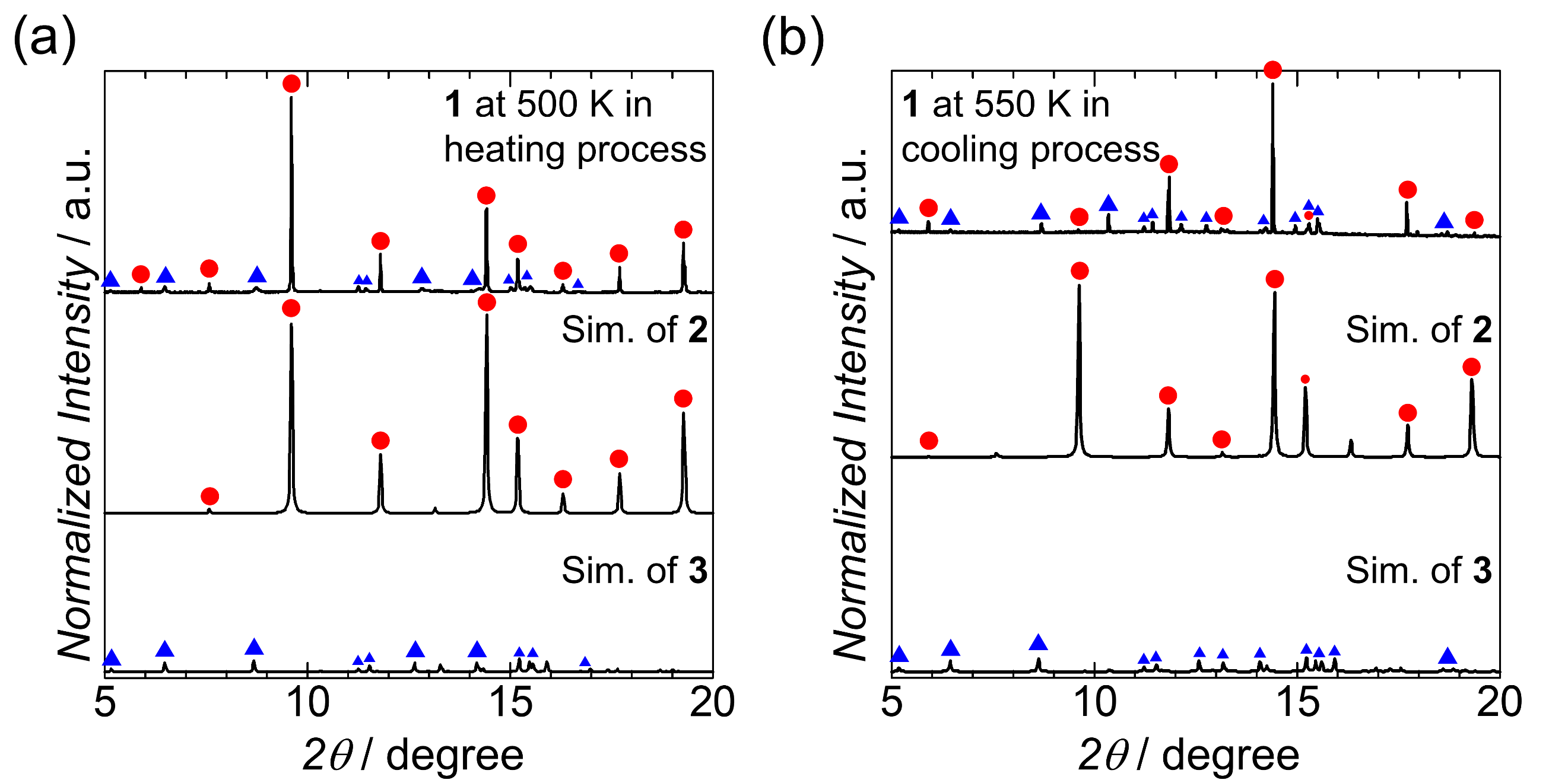


Fig. S18 PXRD pattens of **1** and simulated patterns of **2** and **3** at (a) 500 K in heating process and (b) 550 K in cooling process. These data were recorded on λ = 0.799585 Å. Red circles and blue triangles indicate diffraction peaks of **2** and **3**, respectively.





Fig. S19 VT-IR spectra with **1** as a starting material at 300-570 K.


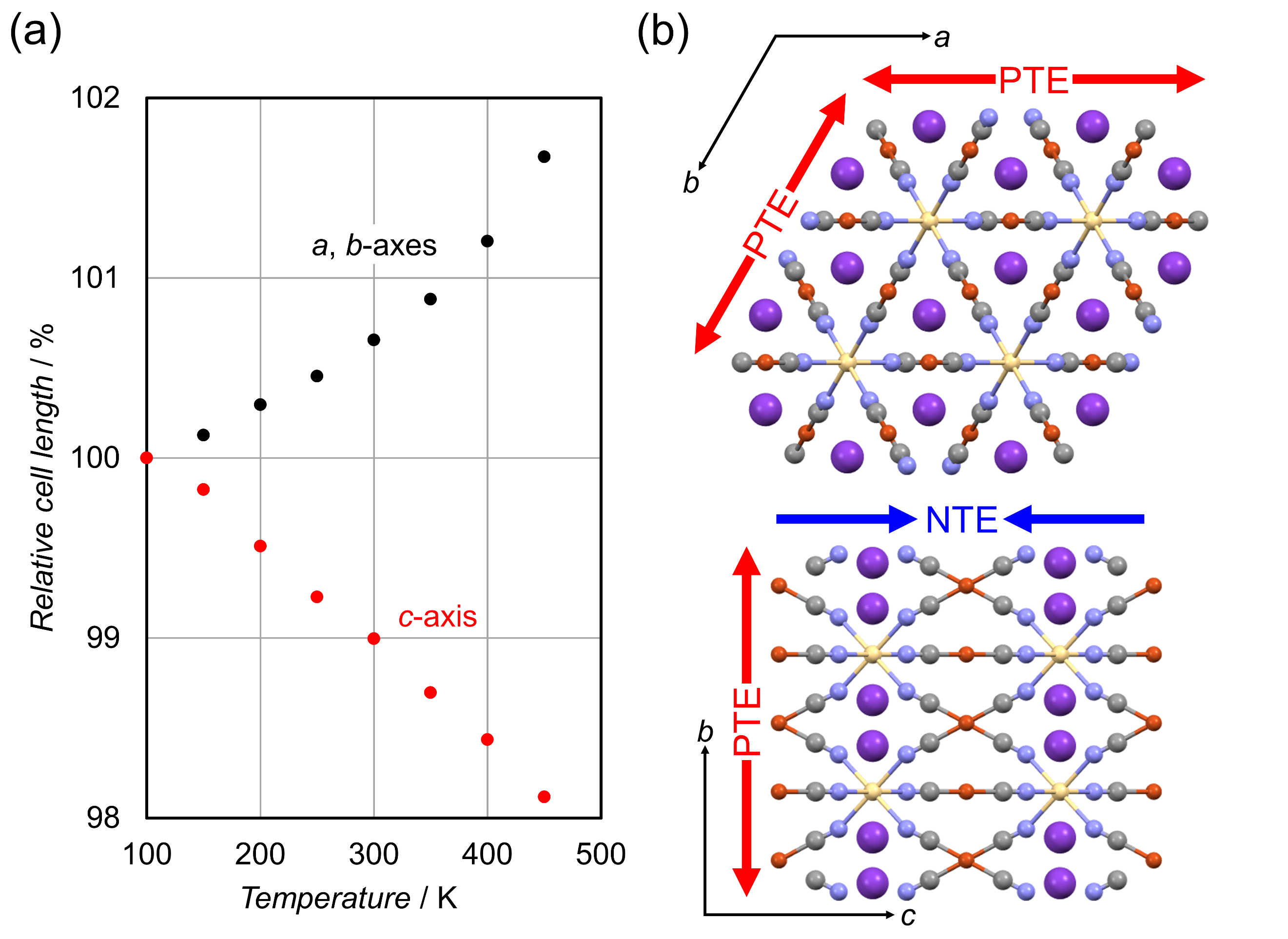


Fig. S20 (a) Relative cell lengths of **2**. (b) Schematic of anisotropic TE behavior of **2**. We found that the presence of [Cu(CN)_2_]^−^ units in the 3D framework of **2** plays an important role in not only its melting but also its thermal expansion (TE) behavior. The structural flexibility of wine-rack-type cyanido-based frameworks is a significant research topic due to the emergence of anisotropic TE. The flexibility of KCd[Ag(CN)_2_]_3_ and KCd[Au(CN)_2_]_3_ is strongly enhanced by the Ag–Ag and Au–Au interactions of the dicyanometallate units within the lattice, resulting in significant anisotropic TE (Figure S20, tables S6, and S7). **2** exhibited anisotropic TE with the same trend as the Ag and Au analogs, but the linear coefficients of thermal expansion (CTE) in each axis were noticeably smaller than those of the Ag and Au analogs (Table S11). The CTE values were α*_a_*_,_ *_b_* = 41.8 (16) MK^−1^ (M = 10^6^), α*_c_* = −47.1 (6) MK^−1^, and α*_V_* = 35.2 MK^−1^. The resulting decrease in CTEs indicates a lack of significant interactions between Cu centers owing to the small ion size. Thus, the isolation of two-coordinate Cu sites within the lattice decreased the melting point due to geometrical flexibility but uniquely hardened the framework.

Table S6 CTE of **2** and its analogs

| **Compounds** | ***a* (MK^−1^)** | ***c* (MK^−1^)** | **Ref.** |
| --- | --- | --- | --- |
| KCd[Cu(CN)_2_]_3_ (**2**) | 41.8(16) | −47.1(6) | This study |
| KCd[Ag(CN)_2_]_3_ | 74.9–76.6 | −64.3–−65.6 | 44 |
| KMn[Ag(CN)_2_]_3_ | 61(2) | −60(3) | 45 |
| In[Ag(CN)_2_]_3_ | 104–106 | −83–−85 | 44 |
| In[Ag(CN)_2_]_3_∙*n*H_2_O | 95.0–98.0 | −73.0–−75.0 | 44 |
| KCd[Au(CN)_2_]_3_ | 71.4–72.9 | −56.1–−57.1 | 44 |
| KNi[Au(CN)_2_]_3_ | 58.7–59.7 | −56.1–−57.1 | 44 |
| In[Au(CN)_2_]_3_ | 83.9–86.1 | −62.2–−63.3 | 44 |
| Ag_3_[Co(CN)_6_] | 144(9) | −126(4) | 46 |
| Ag_3_[Fe(CN)_6_] | 124.0(10) | −113(3) | 46 |


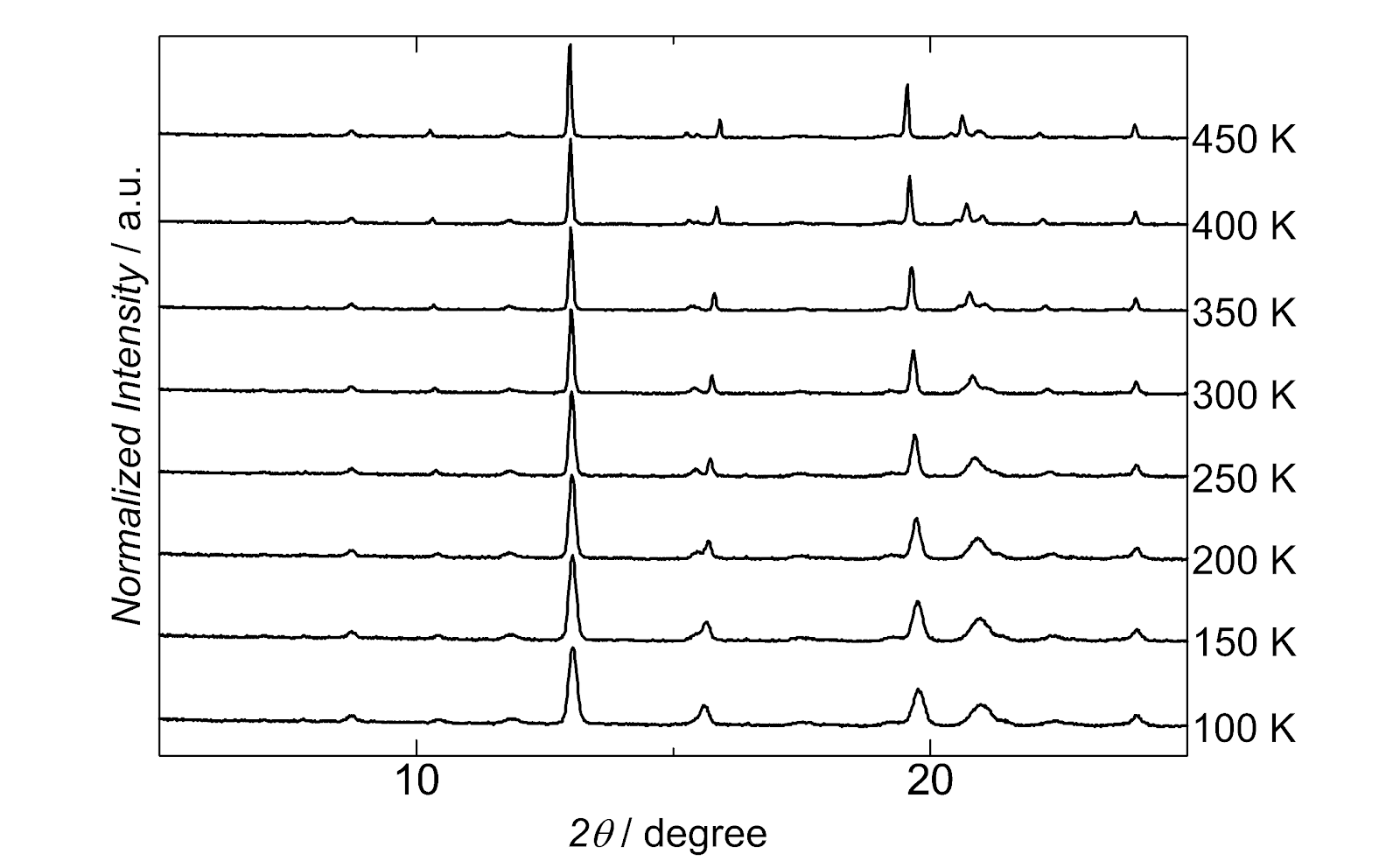


Fig. S21 VT-PXRD for **CdCu_dehyd_** (λ = 1.08 Å)

Table S7 Cell parameters of **2** at 100 K–450 K

| **Temp. (K)** | ***a* and *b*-axes (Å)** | ***c*-axis (Å)** | **V (Å^3^)** |
| --- | --- | --- | --- |
| **100** | 6.863(1) | 7.967(3) | 325.011(5) |
| **150** | 6.872(1) | 7.953(2) | 325.244(4) |
| **200** | 6.884(1) | 7.928(2) | 325.314(4) |
| **250** | 6.894(1) | 7.901(2) | 325.427(4) |
| **300** | 6.908(1) | 7.887(1) | 325.967(3) |
| **350** | 6.924(1) | 7.863(1) | 326.440(3) |
| **400** | 6.946(1) | 7.842(2) | 327.660(4) |
| **450** | 6.978(2) | 7.817(3) | 329.592(7) |


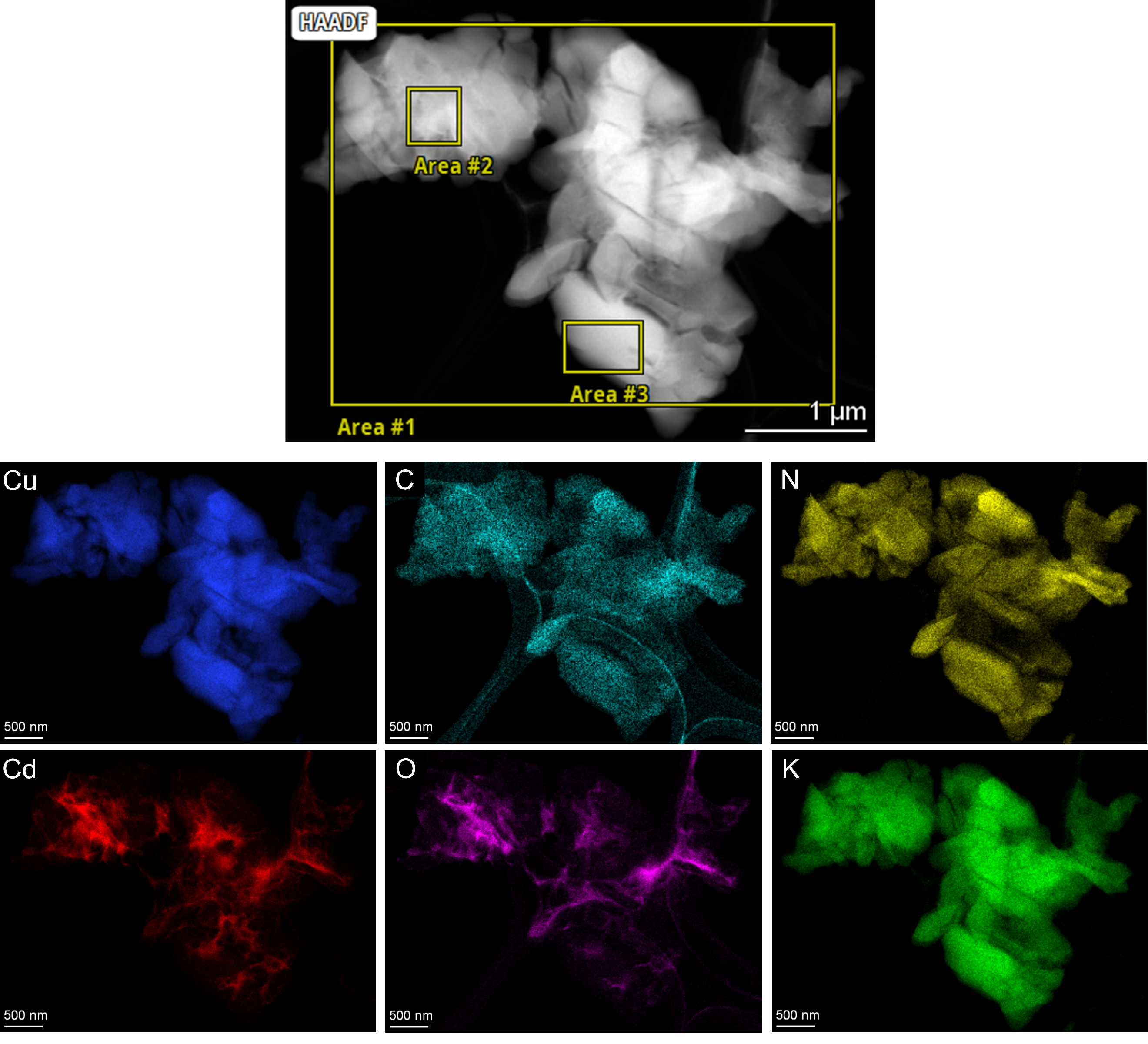


Fig. S22 TEM image and elemental mapping of the recrystallized sample after melting. Areas 1, 2, and 3 are consistent with the data presented in Table S8

Table S8 Elemental ratio (atomic %) of Areas 1, 2, and 3 in Fig. S21. Areas 1 and 2 include high C content due to the overlaps with the carbon film

| Element | Area 1 | Area 2 | Area 3 |
| --- | --- | --- | --- |
| C | 19.13 ± 1.38 | 20.15 ± 1.29 | 15.21 ± 1.30 |
| N | 17.34 ± 3.07 | 13.64 ± 2.48 | 18.94 ± 3.36 |
| O | 5.54 ± 1.12 | 16.05 ± 2.83 | 1.26 ± 0.27 |
| K | 18.89 ± 2.96 | 16.62 ± 2.61 | 20.85 ± 3.28 |
| Cu | 36.00 ± 3.75 | 23.72 ± 2.88 | 42.94 ± 4.19 |
| Cd | 3.10 ± 0.41 | 9.82 ± 1.19 | 0.79 ± 0.11 |
| Assumed formula |  | KCd[Cu(CN)_2_]_3_  CdO, Cu_3_N | K_2_Cu_3_(CN)_5_ |


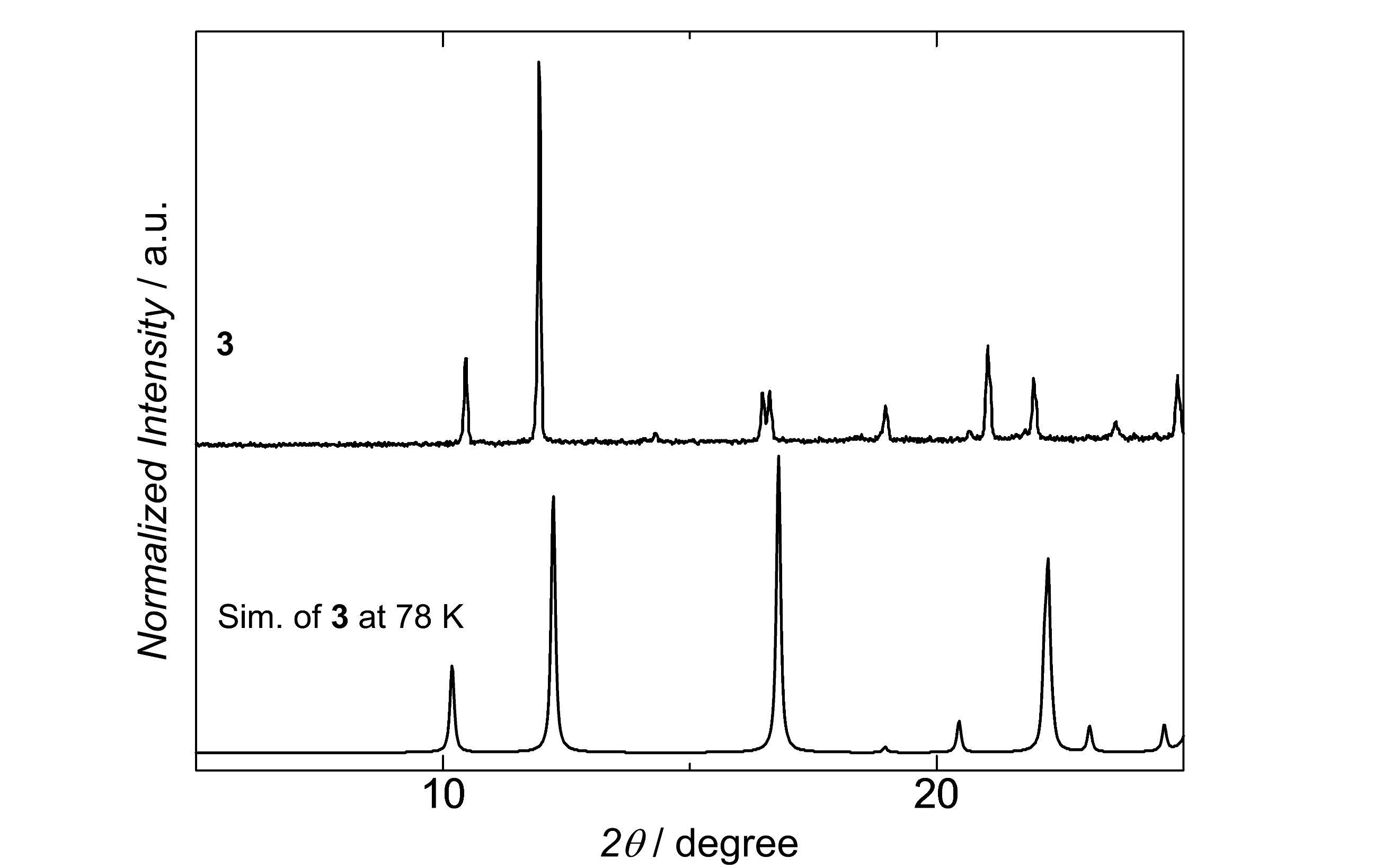


Fig. S23 PXRD pattern of directly synthesized **3**, simulated pattern of **3** at 78 K. This data was recorded on λ = 1.54 Å.


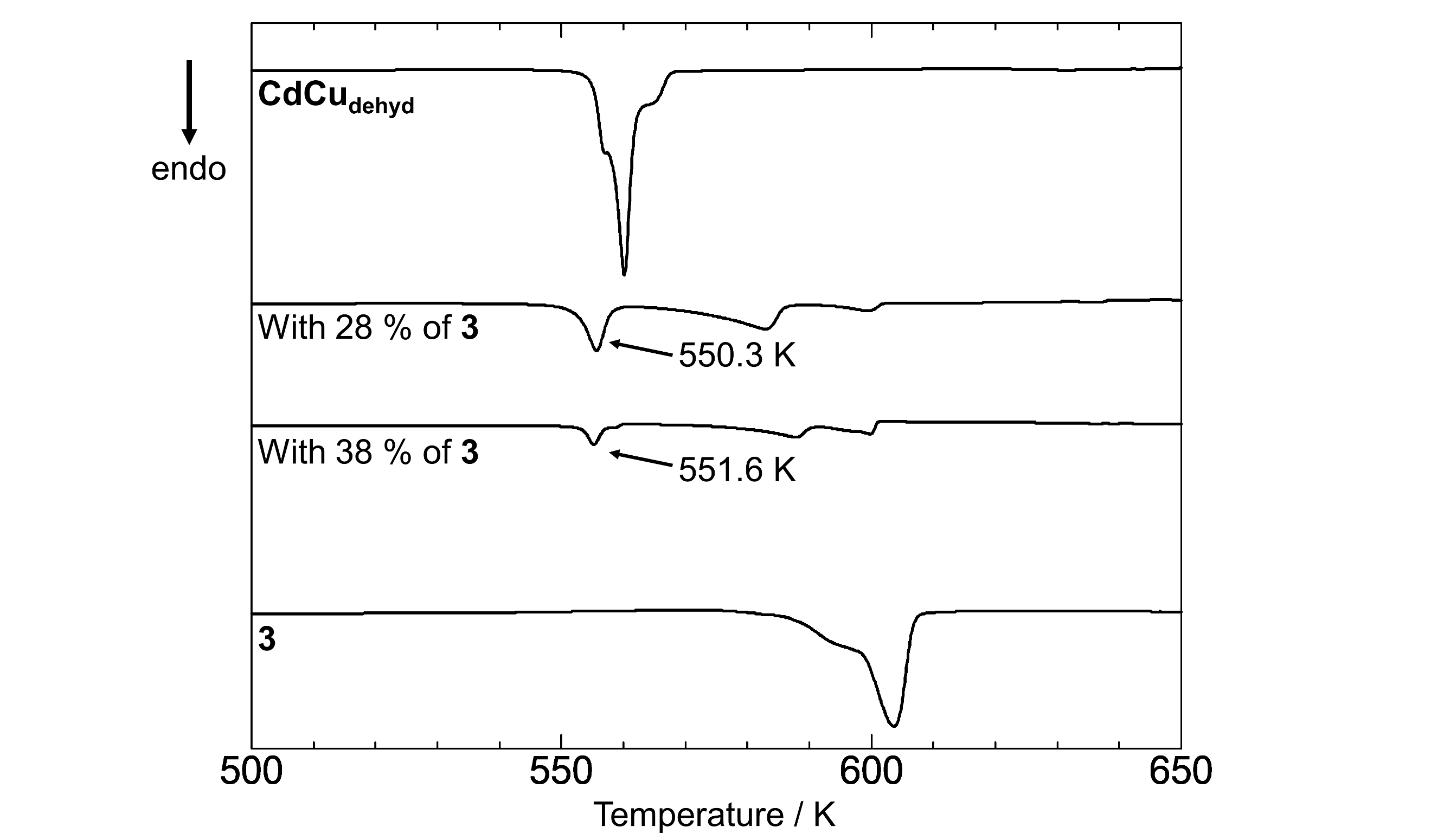


Fig. S24 DSC curves of **CdCu_dehyd_**, **3**, and powder mixtures in heating process up to 773 K. Note that powder mixing was conducted with a mortar. It was demonstrated that **CdCu_dehyd_** exhibited no peak around 600 K although it contained **3**. On the other hand, small peaks around 600 K in the DSC curves of the mixures indicate that a small amount of **3** remains. However, increasing the mixing ratio led to a decrease followed by an increase in the melting point of **CdCu_dehyd_**. This behavior is consistent with the higher melting point of **3** and indicates that the melting temperature is determined by a delicate balance between interfacial interactions and intrinsic thermodynamic properties.

Table S9 Melting points of **CdCu_dehyd_**, and powder mixtures of **CdCu_dehyd_** and **3**.

| Ratio of **CdCu_dehyd_** (wt%) | Ratio of **3** (wt%) | Melting point (K) |
| --- | --- | --- |
| 100 | 0 | 559.9 |
| 72 | 28 | 550.3 |
| 62 | 38 | 551.6 |


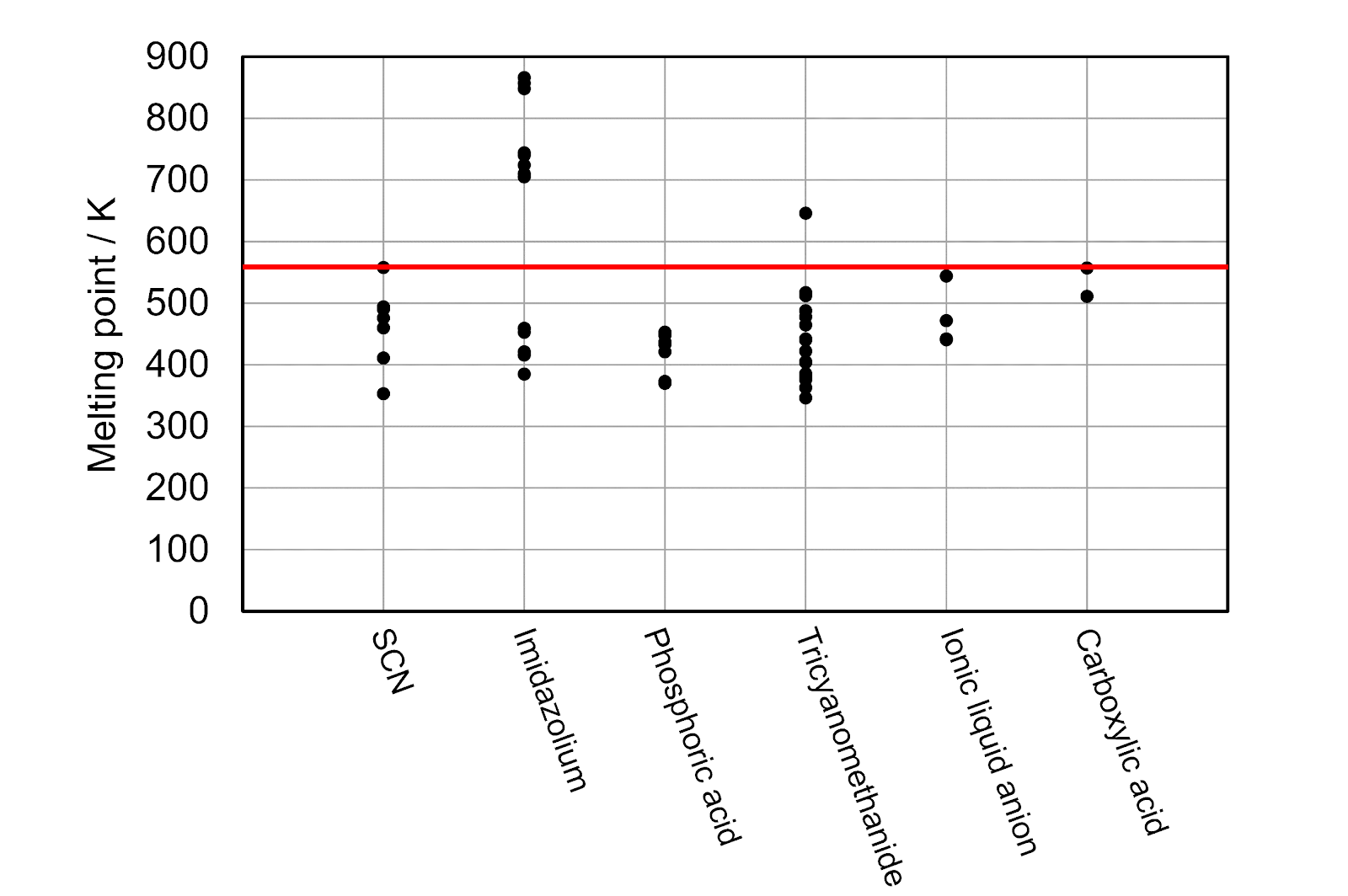


Fig. S25 Melting points of meltable metal–organic framework (MOF)/coordination polymer (CP) consisting of various components.^22-41^ These groups include each analog. The red line indicates the melting points of **2** and **3**


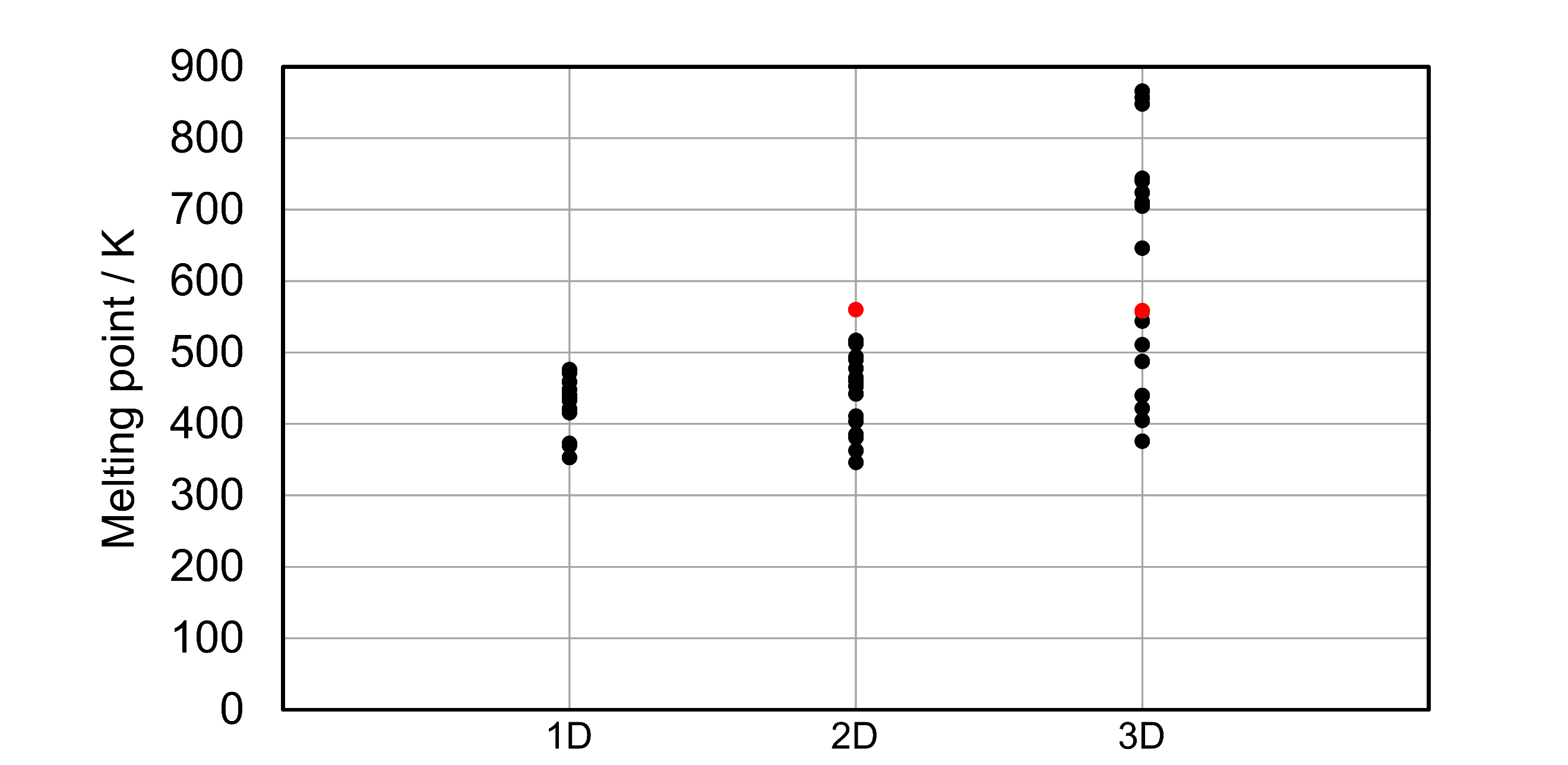


Fig. S26 Melting points of meltable MOF/CP categorized by structural dimensionality.^22-41^ The red point indicates **2** and **3**

Table S10 Melting point and structural dimensionality of representative meltable MOF/CP

| **Compounds** | **Dimensionality** | **Melting point (K)** | **reference** |
| --- | --- | --- | --- |
| Zn(Im)_2_ (ZIF-4) | 3D | 866 | 23 |
| Zn(Im)_1.75_(bIm)_0.25_(ZIF-62) | 3D | 710 | 24 |
| [Zn(HPO_4_)(H_2_PO_4_)_2_]∙2H_2_Im | 1D | 433 | 42 |
| Zn(H_2_PO_4_)_2_(HTr)_2_ | 2D | 453 | 38 |
| Zn(SCN) | 2D | 494 | 40 |
| Cu_2_(SCN)_3_(C2bpy) | 2D | 460 | 33 |
| [Ru(Cp)(C_6_H_6_)][K(TCM)_2_] | 2D | 512.3 | 30 |
| [PrPy][K(TCM)_2_] | 2D | 362.9 | 32 |
| Mg_4_(adipate)_4_(DMA)(H_2_O) | 3D | 557 | 22 |
| Mn_4_(adipate)_4_(DMA)(H_2_O) | 3D | 511 | 22 |
| Cu(TFSI)_2_(bpp)_2_ | 1D | 471.6 | 43 |
| Cu(Ms_2_N)_2_(bpp)_2_ | 1D | 440.6 | 43 |
| **2** | 3D | 559.9 | This work |
| **3** | 2D | 556.9 | This work |

Im^+^: Imidazolium; bIm^+^: benzimidazolium; HTr: 1,2,4-triazole; C2bpy^+^: 1-ethyl-[4,4′-bipyridin]-1-ium; Cp: cyclopentadiene; TCM^−^: tricyanomethanide; DMA: N,N′-dimethylacetamide; TFSI^−^: bis(trifluoromethylsulfonyl)imide; bpp: 1,3-bis(4-pyridyl)propane; Ms_2_N^−^: bis(methanelsulfonyl)imide


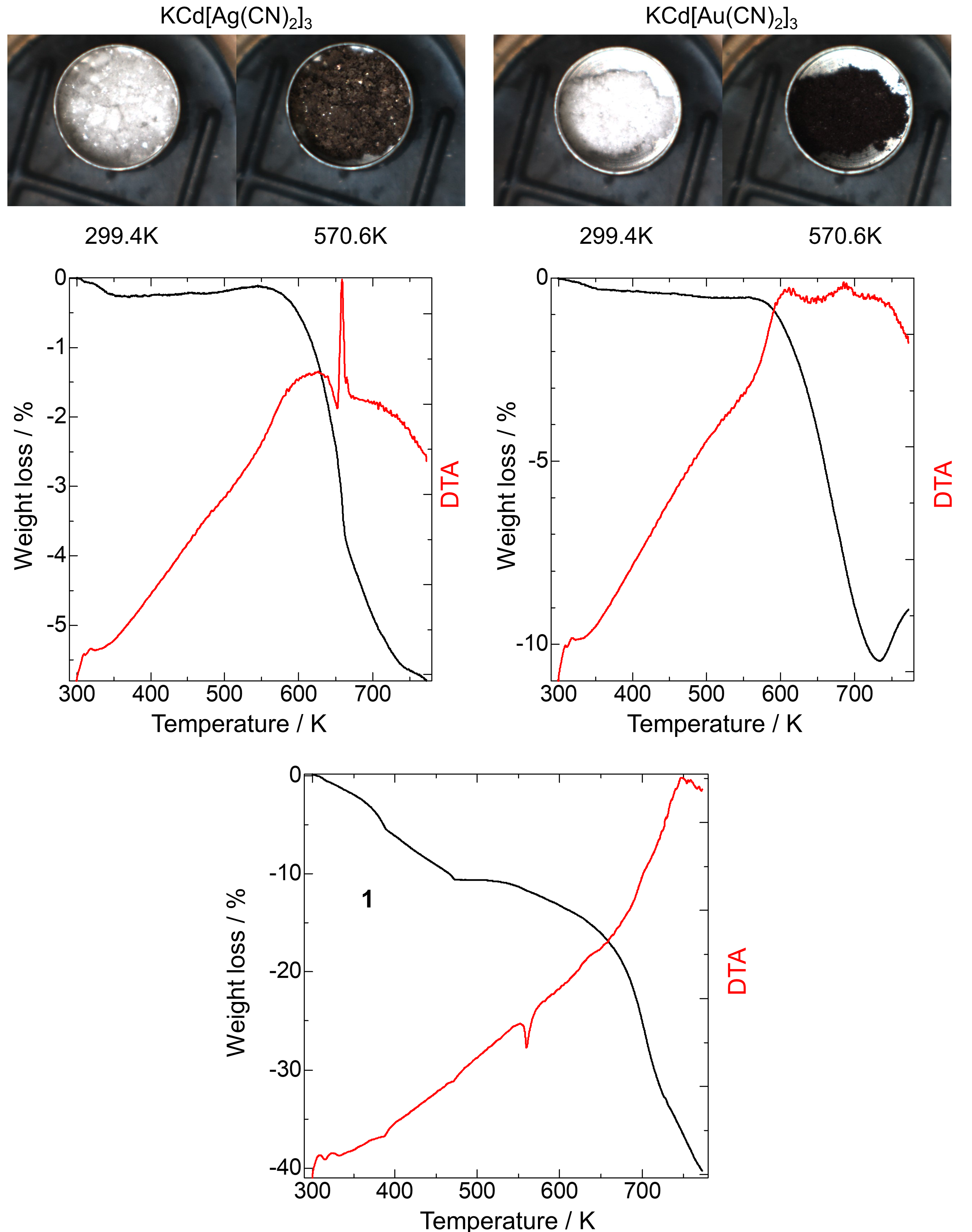


Fig. S27 Sample pictures and thermogravimetry (TG)–differential thermal analysis (DTA) results for KCd[Ag(CN)_2_]_3_ and KCd[Ag(CN)_2_]_3_. The images were taken at 299.4 and 570.6 K. The TG-DTA result of **1** is also shown


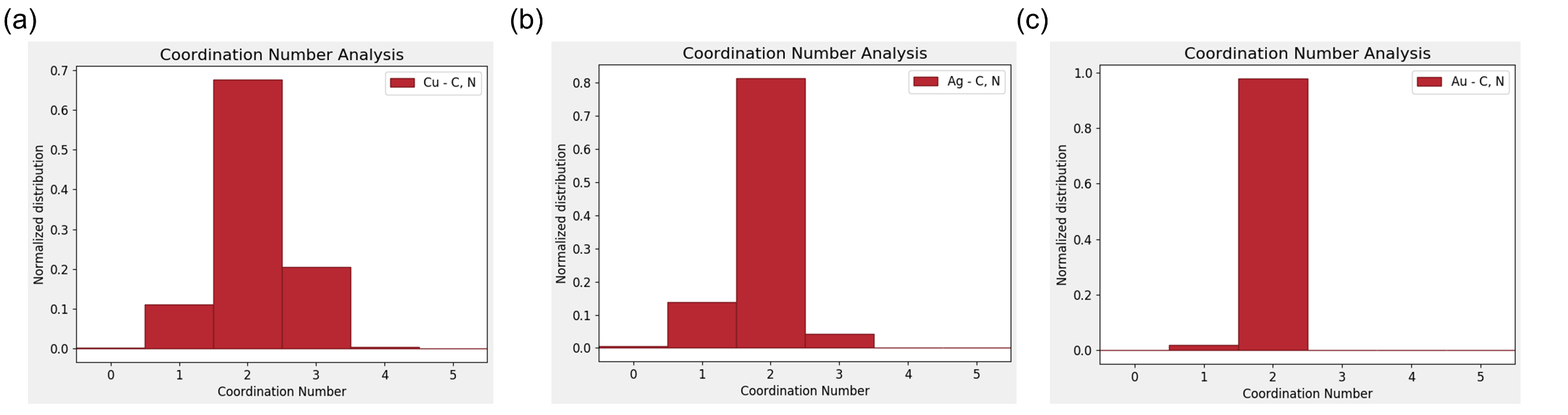


Fig. S28 Distribution ratios of coordination numbers for (a) Cu in 2, (b) Ag in KCd[Ag(CN)₂]₃, and (c) Au in KCd[Au(CN)₂]₃ obtained from MD simulations at 1000 K. Coordination numbers were counted over 1000–1500 fs from the beginning of supplemental movies, using a threshold distance of +10% of the sum of van der Waals radii. A noticeable increase in coordination numbers above three for Cu indicates the labile nature of its two-coordinate environment.


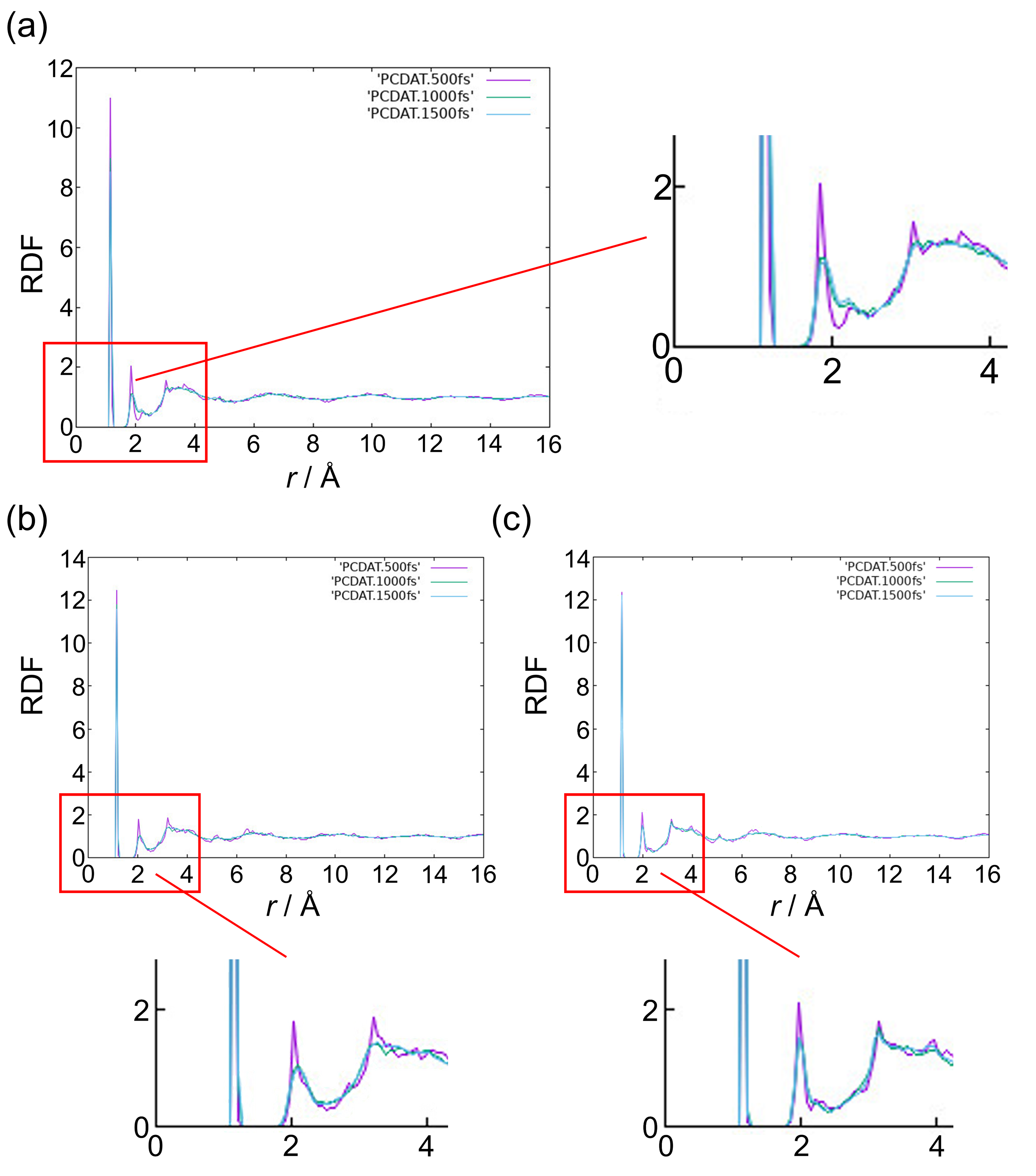


Fig. S29 Simulated radial distribution function (RDF) of (a) **2**, (b) KCd[Ag(CN)_2_]_3_, and (c) KCd[Au(CN)_2_]_3_. The RDF curves indicated by the purple, green, and blue lines were calculated using trajectories from the intervals of 0–500, 500–1,000, and 1,000–1,500 at 1,000 K, respectively

Table S11 Enthalpy of formation of **2**, KCd[Ag(CN)_2_]_3_, and KCd[Au(CN)_2_]_3_. These values were calculated for 2 × 2 × 2 supercell

| Compound | Enthalpy of formation (eV/f.u.) |
| --- | --- |
| **2** | 3.88 |
| KCd[Ag(CN)_2_]_3_ | 4.72 |
| KCd[Au(CN)_2_]_3_ | 3.26 |

(a)


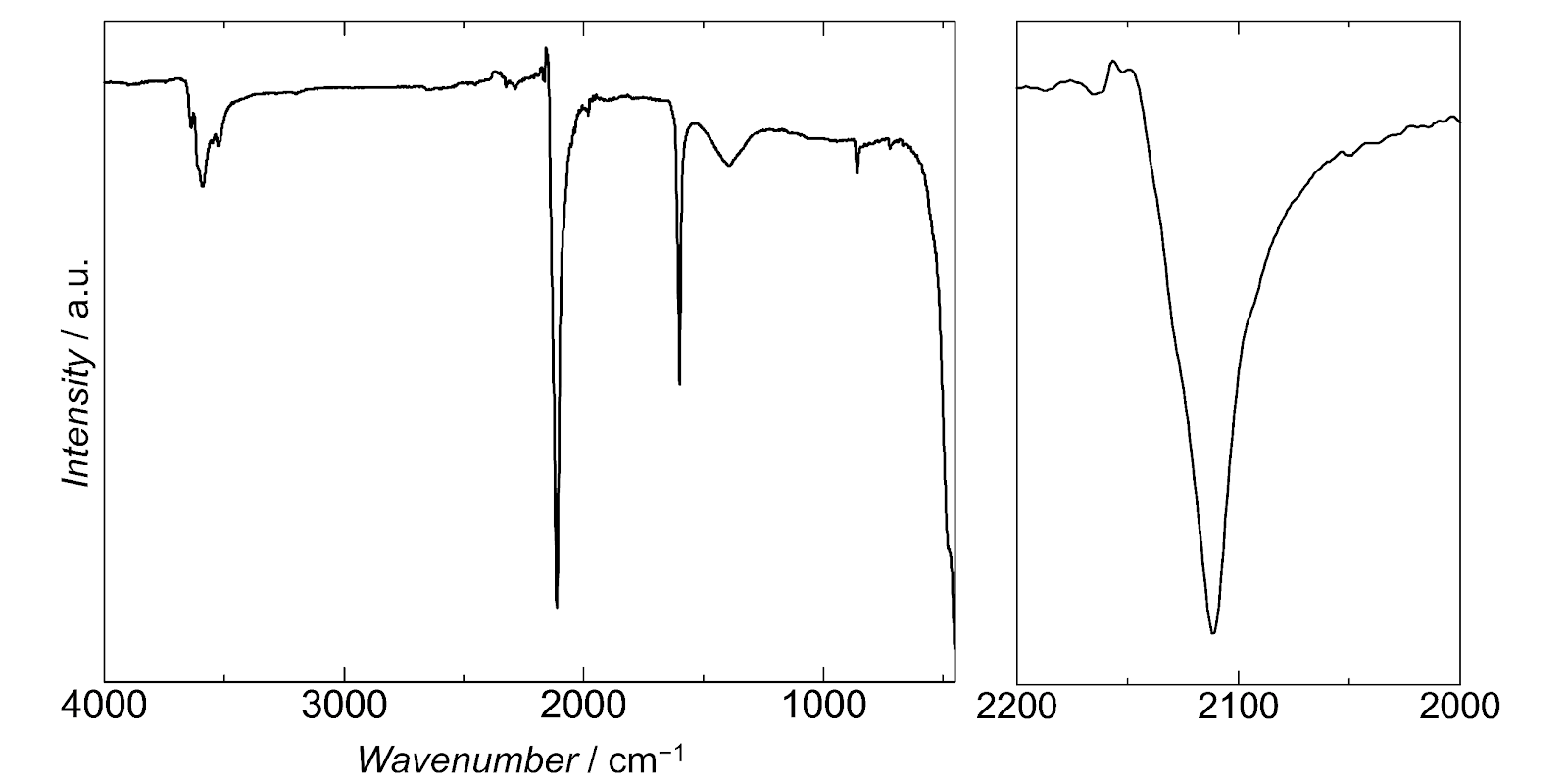


(b)


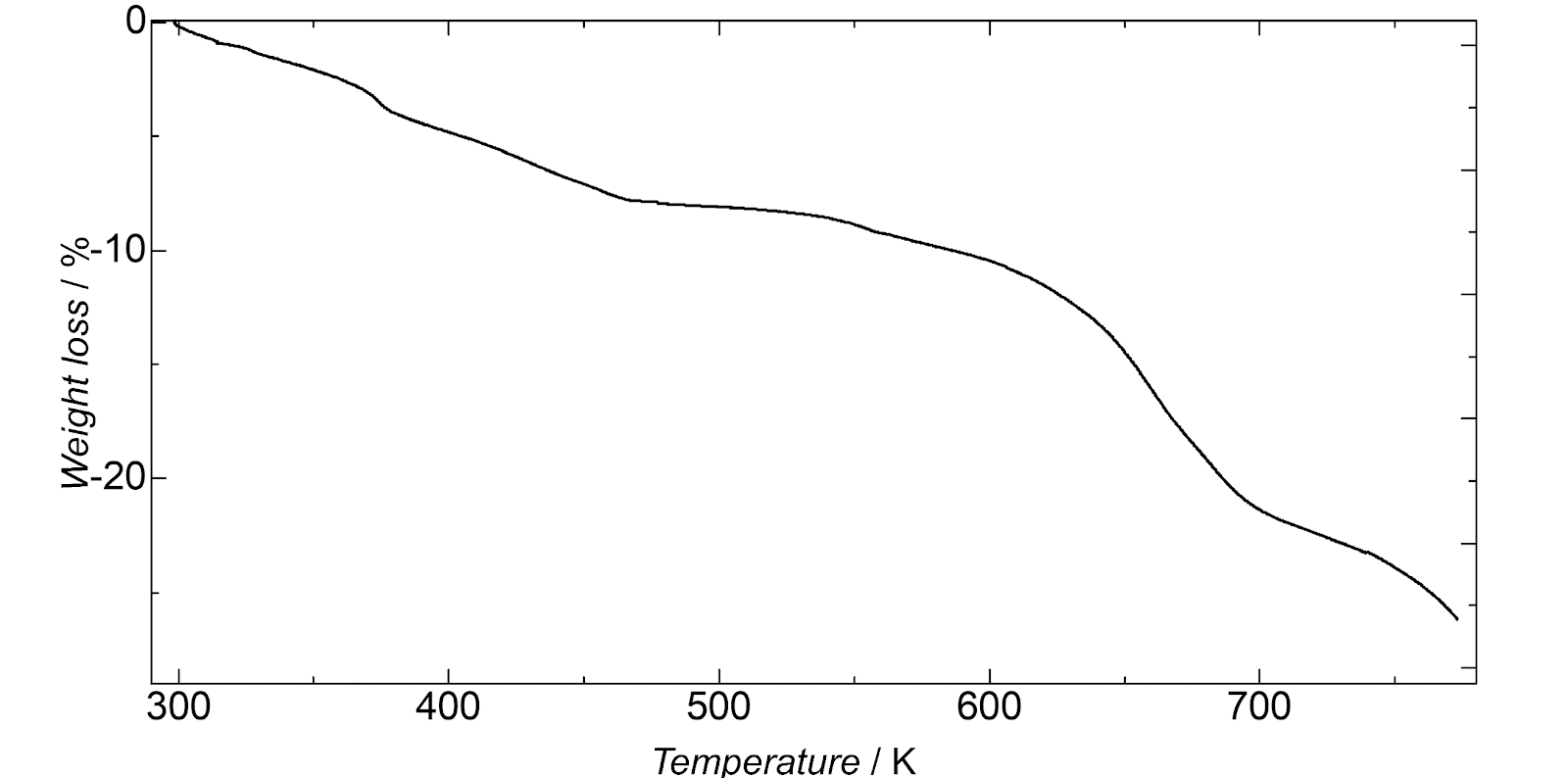


Fig. S30 (a) IR spectra and (b) TGA curve of **CdCu_rehyd_**


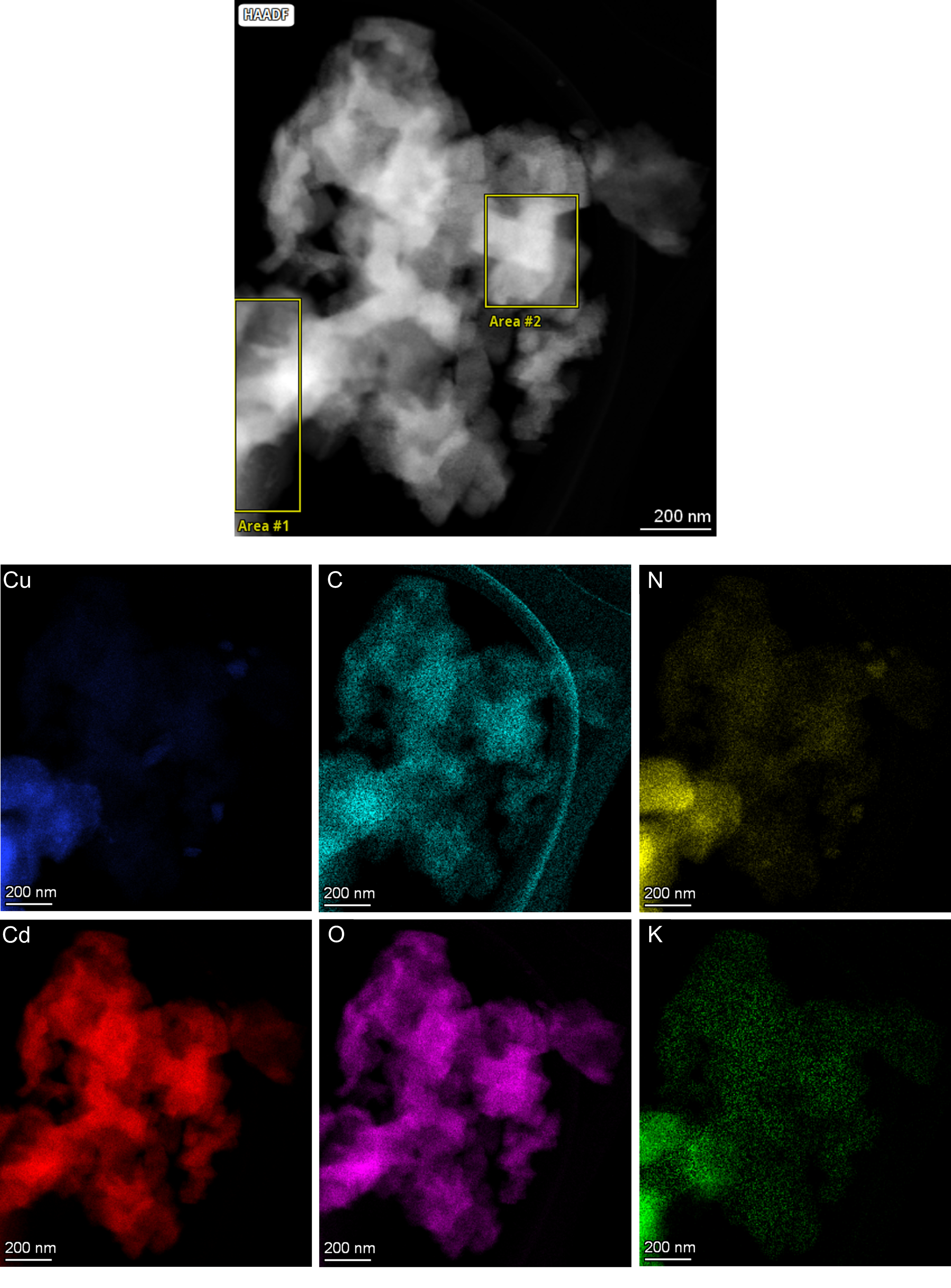


Fig. S31 TEM image and elemental mapping of **CdCu_rehyd_**. Areas 1 and 2 are consistent with the data presented in Table S12

Table S12 Elemental ratio (atomic %) of Areas 1 and 2 in Fig. S31

| **Element** | Area 1 | Area 2 |
| --- | --- | --- |
| **C** | 21.71 ± 1.56 | 21.02 ± 2.18 |
| **N** | 17.26 ± 3.05 | 2.93 ± 0.64 |
| **O** | 28.06 ± 4.22 | 49.53 ± 5.20 |
| **K** | 4.35 ± 0.80 | 0.39 ± 0.13 |
| **Cu** | 13.84 ± 1.96 | 1.99 ± 0.35 |
| **Cd** | 14.79 ± 1.79 | 24.14 ± 3.20 |
| **Assumed formula** | Cd(H_2_O)_2_Cu_4_(CN)_8_  CdO | CdO |


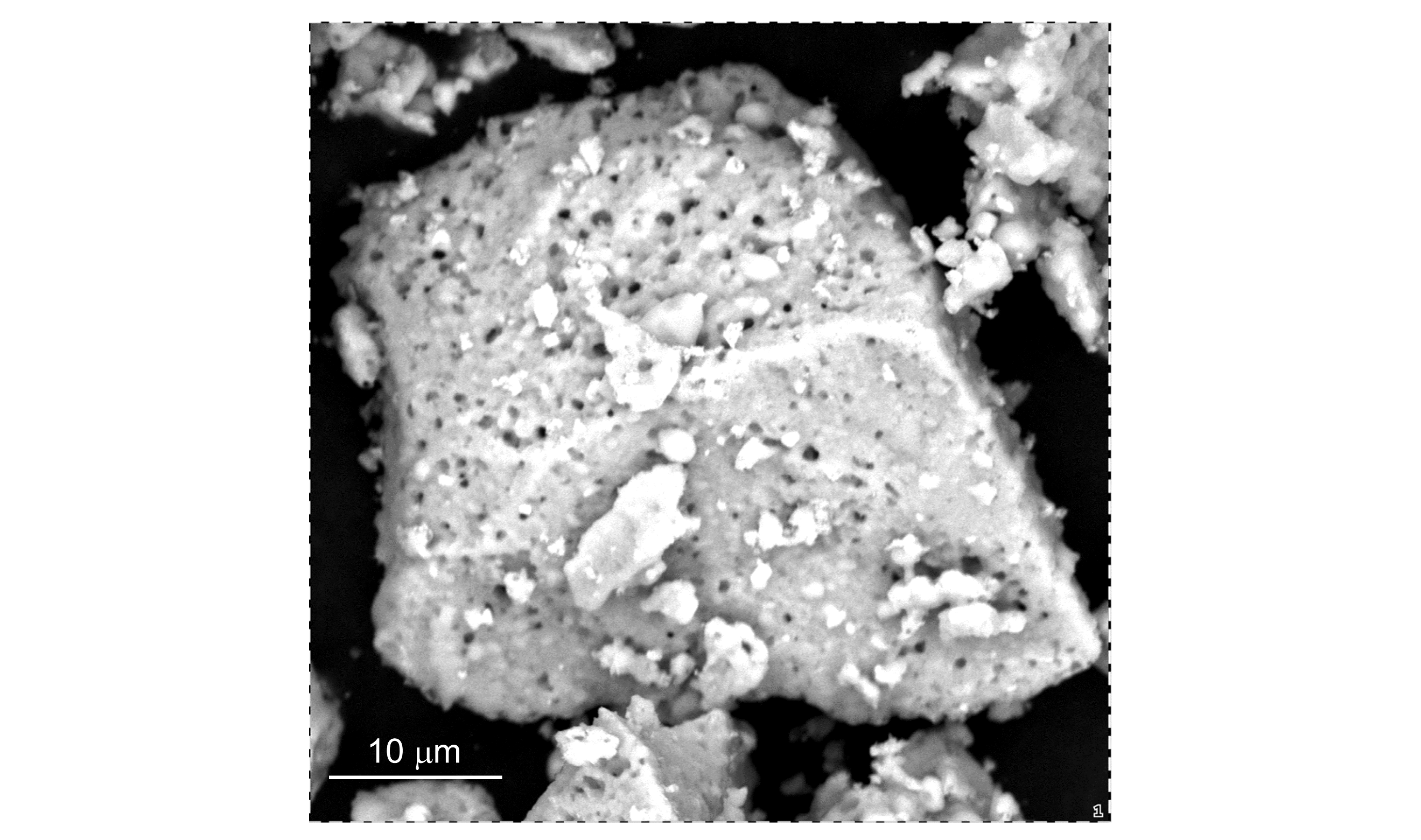


Fig. S32 SEM image of **CdCu_rehyd_**. There are many small holes on the surface as **CdCu_dehyd_**.

1. A. H. Pohl, A. M. Chippindale, S. J. Hibble, “New copper (I) cyanide networks: Interpenetration, self-penetration and polymorphism.” *Solid State Sci.* **2006**, *8*, 379.
2. O. V. Dolomanov, L. J. Bourhis, R. J. Gildea, J. A. K. Howard, H. Puschmann, “OLEX2: a complete structure solution, refinement and analysis program.” *J. Appl. Crystallogr.* **2009**, *42*, 339.
3. G. M. Sheldrick, “SHELXT - integrated space-group and crystal-structure determination.” *Acta Crystallogr. A Found Adv.* **2015**, *71*, 3.
4. G. M. Sheldrick, “Crystal structure refinement with SHELXL.” *Acta Crystallogr. C Struct. Chem.* **2015**, *71*, 3.
5. S. Kawaguchi, M. Takemoto, K. Osaka, E. Nishibori, C. Moriyoshi, Y. Kubota, Y. Kuroiwa, K. Sugimoto, “High-throughput powder diffraction measurement system consisting of multiple MYTHEN detectors at beamline BL02B2 of SPring-8.” *Rev. Sci. Instrum.* **2017**, *88*, 085111.
6. D. Šišak Jung, “MYTHEN2 detector series.” *J. Appl. Crystallogr.* **2015**, *48*, 2044.
7. D. Gogoi, T. Sasaki, T. Nakane, A. Kawamoto, H. Hojo, G. Kurisu, R. Thakuria, “Structure Elucidation of Olanzapine Molecular Salts by Combining Mechanochemistry and Micro-Electron Diffraction.” *Cryst. Growth Des.* **2023**, *23*, 5821.
8. D. N. Mastronarde, “SerialEM: A Program for Automated Tilt Series Acquisition on Tecnai Microscopes Using Prediction of Specimen Position.” *Microsc. Microanal.* **2003**, *9*, 1182.
9. M. T. B. Clabbers, T. Gruene, J. M. Parkhurst, J. P. Abrahams, D. G. Waterman, “Electron diffraction data processing with DIALS.” *Acta Crystallogr. D Struct. Biol*. **2018**, *74*, 506.
10. G. Winter, D. G. Waterman, J. M. Parkhurst, A. S. Brewster, R. J. Gildea, M. Gerstel, L. Fuentes-Montero, M. Vollmar, T. Michels-Clark, I. D. Young, N. K. Sauter, G. Evans, “DIALS: implementation and evaluation of a new integration package.” *Acta Crystallogr. D Struct. Biol.* **2018**, *74*, 85.
11. O. Tange, in *The USENIX Magazine* **2011**, Vol. 36, 42.
12. J. Hattne, M. W. Martynowycz, P. A. Penczek, T. Gonen, “MicroED with the Falcon III direct electron detector.” *IUCrJ* **2019**, *6*, 921.
13. R. J. Gildea, J. Beilsten-Edmands, D. Axford, S. Horrell, P. Aller, J. Sandy, J. Sanchez-Weatherby, C. D. Owen, P. Lukacik, C. Strain-Damerell, R. L. Owen, M. A. Walsh, G. Winter, “xia2.multiplex: a multi-crystal data-analysis pipeline.” *Acta Crystallogr. D Struct. Biol.* **2022**, *78*, 752.
14. J. Beilsten-Edmands, G. Winter, R. Gildea, J. Parkhurst, D. Waterman, G. Evans, “Scaling diffraction data in the DIALS software package: algorithms and new approaches for multi-crystal scaling.” *Acta Crystallogr. D Struct. Biol.* **2020**, *76*, 385.
15. T. R. Schneider, G. M. Sheldrick, “Substructure solution with *SHELXD*.” *Acta Crystallogr. D Struct. Biol.* **2002**, *D58*, 1772.
16. G. Kresse, “Efficient iterative schemes for ab initio total-energy calculations using a plane-wave basis set.” *Phys. Rev. B: Condens. Matter Phys.* **1996**, *54*, 169.
17. G. Kresse, J. Furthmiiller, “Efficiency of ab-initio total energy calculations for metals and semiconductors using a plane-wave basis set.” *Comput. Mater. Sci.* **1996**, *6*, 15.
18. J. P. Perdew, K. Burke, M. Ernzerhof, “Generalized Gradient Approximation Made Simple.” *Phys. Rev. Lett.* **1996**, *77*, 3865.
19. G. Kresse, D. Joubert, “From ultrasoft pseudopotentials to the projector augmented-wave method.” *Phys. Rev. B: Condens. Matter Phys.* **1999**, *59*, 1758.
20. S. Grimme, S. Ehrlich, L. Goerigk, “Effect of the damping function in dispersion corrected density functional theory.” *J. Comput. Chem.* **2011**, *32*, 1456.
21. D. J. Evans, B. L. Holian, “The Nose–Hoover thermostat.” *J. Chem. Phys.* **1985**, *83*, 4069.
22. M. Kim, H. S. Lee, D. H. Seo, S. J. Cho, E. C. Jeon, H. R. Moon, “Melt-quenched carboxylate metal-organic framework glasses.” *Nat. Commun.* **2024**, *15*, 1174.
23. T. D. Bennett, J. C. Tan, Y. Yue E. Baxter, C. Ducati, N. J. Terrill, H. H. Yeung, Z. Zhou, W. Chen, S. Henke, A. K. Cheetham, G. N. Greaves, “Hybrid glasses from strong and fragile metal-organic framework liquids.” *Nat. Commun.* **2015**, *6*, 8079.
24. T. D. Bennett, Y. Yue, P. Li, A. Qiao, H. Tao, N. G. Greaves, T. Richards, G. I. Lampronti, S. A. Redfern, F. Blanc, O. K. Farha, J. T. Hupp, A. K. Cheetham, D. A. Keen, “Melt-Quenched Glasses of Metal-Organic Frameworks.” *J. Am. Chem. Soc.* **2016**, *138*, 3484.
25. A. M. Bumstead, M. L. Ríos Gómez, M. F. Thorne, A. F. Sapnik, L. Longley, J. M. Tuffnell, D. S. Keeble, D. A. Keen, T. D. Bennett, “Investigating the melting behaviour of polymorphic zeolitic imidazolate frameworks.” *CrystEngComm* **2020**, *22*, 3627.
26. C. Das, S. Horike, “Crystal melting and vitrification behaviors of a three-dimensional nitrile-based metal-organic framework.” *Faraday Discuss.* **2021**, *225*, 403.
27. C. Das, T. Ogawa, S. Horike, “Stable melt formation of 2D nitrile-based coordination polymer and hierarchical crystal-glass structuring.” *Chem. Commun.* **2020**, *56*, 8980.
28. L. Frentzel-Beyme, M. Kloss, P. Kolodzeiski, R. Pallach, S. Henke, “Meltable Mixed-Linker Zeolitic Imidazolate Frameworks and Their Microporous Glasses: From Melting Point Engineering to Selective Hydrocarbon Sorption.” *J. Am. Chem. Soc.* **2019**, *141*, 12362.
29. M. Inukai, Y. Nishiyama, K. Honjo, C. Das, S. Kitagawa, S. Horike, “Glass-phase coordination polymer displaying proton conductivity and guest-accessible porosity.” *Chem. Commun.* **2019**, *55*, 8528.
30. H. Kimata, T. Mochida, T. “Crystal Structures and Melting Behaviors of 2D and 3D Anionic Coordination Polymers Containing Organometallic Ionic Liquid Components.” *Chem. Eur. J.* **2019**, *25*, 10111.
31. T. Mochida, Y. Qiu, Y. Funasako, M. Inokuchi, M. Noguchi, H. Fujimori, Y. Furushima, “Ionic liquid-containing coordination polymer: solvent-free synthesis, incongruent melting, and glass formation.” *Chem. Commun.* **2022**, *58*, 6725.
32. T. Mochida, Y. Qiu, R. Sumitani, H. Kimata, Y. Furushima, “Incongruent Melting and Vitrification Behaviors of Anionic Coordination Polymers Incorporating Ionic Liquid Cations.” *Inorg. Chem.* **2022**, *61*, 14368.
33. S. S. Nagarkar, H. Kurasho, N. T. Duong, Y. Nishiyama, S. Kitagawa, S. Horike, “Crystal melting and glass formation in copper thiocyanate based coordination polymers.” *Chem. Commun.* **2019**, *55*, 5455.
34. E. T. Spielberg, E. Edengeiser, B. Mallick, M. Havenith, A. V. Mudring, “(1-Butyl-4-methyl-pyridinium)[Cu(SCN)_2_]: a coordination polymer and ionic liquid.” *Chem. Eur. J.* **2014**, *20*, 5338.
35. Y.-J. Su, Y.-L. Cui, Y. Wang, R.-B. Lin, W.-X. Zhang, J.-P. Zhang, X.-M. Chen, “Copper(I) 2-Isopropylimidazolate: Supramolecular Isomerism, Isomerization, and Luminescent Properties.” *Cryst. Growth Des.* **2015**, *15*, 1735.
36. H. Tao, T. D. Bennett, Y. Yue, “Melt-Quenched Hybrid Glasses from Metal-Organic Frameworks.” *Adv. Mater.* **2017**, *29*, 1601705.
37. T. Tiyawarakul, T. Imyen, K. Kongpatpanich, T. Watcharatpong, S. Horike, “Macroscopic shaping of coordination polymer via crystal–glass phase transformation as monolithic catalyst for efficient catalyst recovery.” *APL Materials* **2023**, *11*, 0144603.
38. D. Umeyama, S. Horike, M. Inukai, T. Itakura, S. Kitagawa, “Inherent proton conduction in a 2D coordination framework.” *J. Am. Chem. Soc.* **2012**, *134*, 12780.
39. D. Umeyama, S. Horike, M. Inukai, T. Itakura, S. Kitagawa, “Reversible solid-to-liquid phase transition of coordination polymer crystals.” *J. Am. Chem. Soc.* **2015**, *137*, 864.
40. C. Wechwithayakhlung, S. Wannapaiboon, S. Na-Phattalung, P. Narabadeesuphakorn, S. Tanjindaprateep, S. Waiprasoet, T. Imyen, S. Horike, P. Pattanasattayavong, “Mixed-Metal Cu-Zn Thiocyanate Coordination Polymers with Melting Behavior, Glass Transition, and Tunable Electronic Properties.” *Inorg. Chem.* **2021**, *60*, 16149.
41. C. Zhou, L. Longley, A. Krajnc, G. J. Smales, A. Qiao, I. Erucar, C. M. Doherty, A. W. Thornton, A. J. Hill, C. W. Ashling, O. T. Qazvini, S. J. Lee, P. A. Chater, N. J. Terrill, A. J. Smith, Y. Yue, G. Mali, D. A. Keen, S. G. Telfer, T. D. Bennett, “Metal-organic framework glasses with permanent accessible porosity.” *Nat. Commun.* **2018**, *9*, 5042.
42. S. Horike, D. Umeyama, M. Inukai, T. Itakura, S. Kitagawa, “Coordination-Network-Based Ionic Plastic Crystal for Anhydrous Proton Conductivity.” *J. Am. Chem. Soc*., **2012**, *134*, 7612.
43. Y. Ohara, T. Nishiguchi, X. Zheng, S.-i. Noro, D. M. Packwood, S. Horike, “Entropically driven melting of Cu-based 1D coordination polymers.” *Chem. Commun.*, **2024**, *60*, 9833.
44. Korⅽok, J. L.; Katz, M. J.; Leznoff, D. B. Impact of Metallophilicity on “Colossal” Positive and Negative Thermal Expansion in a Series of Isostructural Dicyanometallate Coordination Polymers. *J. Am. Soc. Chem.* **2009**, *131*, 4866-4871.
45. Kamali, K.; Ravi, C.; Ravindran, T. R.; Sarguna, R. M.; Sairam, T. N.; Kaur, G. Linear Compressibility and Thermal Expansion of KMn[Ag(CN)_2_]_3_ Studied by Raman Spectroscopy and First-Principles Calculations. *J. Phys. Chem. C* **2013**, *117*, 25704−25713.
46. Goodwin, A. L.; Calleja, M.; Conterio, M. J.; Dove, M. T.; Evans, J. S. O.; Keen, D. A.; Peters, L.; Tucker, M. G.; Colossal Positive and Negative Thermal Expansion in the Framework Material Ag_3_[Co(CN)_6_]. *Science*, **2008**, *3191*, 794-797.
